# *TTN* and *BAG3* in Cancer Therapy–Related Cardiomyopathy Among Long-Term Survivors of Childhood Cancer

**DOI:** 10.1001/jamanetworkopen.2025.15793

**Published:** 2025-06-20

**Authors:** Achal Neupane, Kateryna Petrykey, Kendrick Li, Jennifer French, Xin Zhou, Jian Wang, Cindy Im, Stephanie B. Dixon, Matthew J. Ehrhardt, Daniel A. Mulrooney, John L. Jefferies, M. Monica Gramatges, Eric J. Chow, Smita Bhatia, Leslie L. Robison, Kirsten K. Ness, Melissa M. Hudson, Paul W. Burridge, Gregory T. Armstrong, Yutaka Yasui, Yadav Sapkota

**Affiliations:** 1 Department of Epidemiology and Cancer Control, St Jude Children’s Research Hospital, Memphis, Tennessee; 2 Department of Biostatistics, St Jude Children’s Research Hospital, Memphis, Tennessee; 3 Department of Computational Biology, St Jude Children’s Research Hospital, Memphis, Tennessee; 4 Department of Pediatrics, University of Minnesota, Minneapolis; 5 Department of Oncology, St Jude Children’s Research Hospital, Memphis, Tennessee; 6 School of Public Health, University of Memphis, Memphis, Tennessee; 7 Department of Pediatrics, Baylor College of Medicine, Houston, Texas; 8 Fred Hutchinson Cancer Center, Seattle Children’s Hospital, University of Washington, Seattle; 9 Institute for Cancer Outcomes and Survivorship, University of Alabama at Birmingham; 10 Department of Pharmacology, Northwestern University, Chicago, Illinois

## Abstract

**Importance:**

Cancer therapy–related cardiomyopathy (CCM) is an important concern for childhood cancer survivors. In the general population, rare variants in *TTN* and *BAG3* are associated with an increased risk of familial dilated cardiomyopathy, and common variants are associated with a decreased risk of sporadic dilated cardiomyopathy.

**Objectives:**

To examine associations of common and rare protein-altering variants (PAVs) in *TTN* and *BAG3* with late-onset CCM risk in childhood cancer survivors.

**Design, Setting, and Participants:**

This retrospective cohort study with a prospective follow-up included childhood cancer survivors from the St Jude Lifetime Cohort (SJLIFE) and the Childhood Cancer Survivor Study (CCSS) with prior exposure to anthracyclines and/or chest-directed radiation. Cancer therapy–related cardiomyopathy was clinically assessed in SJLIFE and self-reported in CCSS, with severity graded using Common Terminology Criteria for Adverse Events, version 4.03. The data analysis was conducted from January 4, 2023, to March 6, 2025.

**Exposure:**

Late-onset CCM.

**Main Outcome and Measures:**

Multivariable logistic regression was used to evaluate the association of common variants in *TTN* and *BAG3* with late-onset CCM risk, adjusting for relevant demographic and cancer treatment exposures. In SJLIFE alone, 7 echocardiographic parameters were assessed. Rare PAVs were examined using Fisher exact test. Cohort-specific results were combined using meta-analytic approaches.

**Results:**

The cohort included 1843 childhood cancer survivors from SJLIFE (median [IQR] age at CCM diagnosis, 34.9 [28.0-42.3] years; 53.2% male) and 4577 from CCSS (median [IQR] age at CCM diagnosis, 32.0 [23.0-41.0] years; 51.6% female). In the combined sample of European ancestry survivors from SJLIFE (205 with CCM grade ≥2) and CCSS (248 with CCM grade ≥2), common variants rs3829746-C in *TTN* (odds ratio, 0.81; 95% CI, 0.68-0.97) and rs2234962-C in *BAG3* (odds ratio, 0.79; 95% CI, 0.65-0.95) were associated with a decreased risk of late-onset CCM. In SJLIFE African ancestry survivors, no association was observed with either of the common variants. Rare PAVs were not associated with late-onset CCM in European or African ancestry survivors. In European ancestry survivors, both rs3829746-C and rs2234962-C were also associated with reduced left ventricular end-systolic volume (β [SE], −1.90 [0.65] and −2.68 [0.64], respectively) and global longitudinal peak strain (β [SE], −0.31 [0.13] and −0.30 [0.12]) and with increased left ventricular ejection fraction (β [SE], 0.62 [0.27] and 0.86 [0.27], respectively).

**Conclusions and Relevance:**

The findings of this cohort study show that common variants in *TTN* and *BAG3* are associated with a decreased risk of late-onset CCM among childhood cancer survivors, while rare PAVs showed no association.

## Key Points

**Question:**

What is the association of *TTN* and *BAG3* with late-onset cancer therapy–related cardiomyopathy (CCM) among childhood cancer survivors?

**Findings:**

In this cohort study of 6420 childhood cancer survivors, common variants in *TTN* and *BAG3* were associated with decreased late-onset CCM risk among European ancestry survivors, and these variants were associated with improved left ventricular ejection fraction, end-diastolic and end-systolic volumes, and global longitudinal strain. Rare protein-altering variants in these genes showed no association among European and African ancestry survivors.

**Meaning:**

These findings show that common variants in *TTN* and *BAG3* are associated with a reduced risk of late-onset CCM in childhood cancer survivors, whereas rare protein-altering variants show no association.

## Teaser

This cohort study examines associations of common and rare protein-altering variants in *TNN* and *BAG3* with late-onset cancer therapy–related cardiomyopathy risk in adult childhood cancer survivors.

## Introduction

Late-onset cancer therapy–related cardiomyopathy (CCM) is a leading noncancer cause of morbidity and mortality in adult survivors of childhood cancer. Survivors have a 15-fold higher risk for developing cardiomyopathy compared with their siblings.^1^ Risk factors for late-onset CCM include exposures to anthracyclines and chest-directed radiation therapy, along with younger age at primary cancer diagnosis, female sex, and the presence of traditional cardiovascular risk factors.^2^ However, these clinical risk factors alone do not fully account for a substantial proportion of the disease risk.^3^

Several studies have identified common genetic variants associated with the risk of late-onset CCM in childhood cancer survivors.^3,4,5,6^ Rare variants found in familial dilated cardiomyopathy (DCM) genes in the general population have also been associated with increased CCM risk in both adult and childhood cancer survivors.^3^ Specifically, rare protein-altering variants (PAVs) in *TTN* were enriched among 213 patients with cancer and CCM (7.5%) compared with healthy control individuals (0.7%), not patients with cancer without CCM. However, less than 20% of the patients with CCM were childhood cancer survivors, and the mean time from cancer therapy exposure to CCM was only 0.3 years, indicating an early-onset CCM that may have occurred during cancer treatment. Thus, whether these associations with early-onset CCM identified in adult cancer populations are also true among adult survivors of childhood cancer with late-onset CCM requires additional research.

An exome-wide association study including sporadic cases of DCM identified a common (minor allele frequency ≥5%) missense variant in *TTN*, rs3829746-C, associated with reduced risk.^7^ An independent study also found a directionally consistent, though not statistically significant, association of this single-nucleotide variant (SNV) with DCM.^8^ In Genotype-Tissue Expression data,^9^ rs3829746-C is associated with reduced *TTN* expression in the left ventricle and atrial appendage. Additionally, another study identified a common missense variant, rs2234962-C, in *BAG3* that was associated with decreased sporadic DCM risk, while rare variants in the same gene were associated with increased familial DCM risk.^10,11^ The association of rs2234962-C with DCM has been replicated in several association studies,^7,8,12,13,14^ with additional support from a functional study.^15^ However, the potential role of genetic variants in *TTN* and *BAG3* in the risk of late-onset (occurring ≥5 years after childhood cancer diagnosis) CCM among childhood cancer survivors remains unknown.

To explore this possible role, we examined the associations of both common and rare genetic variants in *TTN* and *BAG3* with the risk of late-onset CCM among long-term survivors of childhood cancer from the 2 largest pediatric cancer survivor cohorts in North America: the St Jude Lifetime Cohort (SJLIFE) and the Childhood Cancer Survivor Study (CCSS).

## Methods

### Study Population

This retrospective cohort study with prospective follow-up included participants from SJLIFE (1605 survivors of European ancestry and 238 survivors of African ancestry) and CCSS (4577 survivors of European ancestry). The study methodologies and characteristics of both cohorts have been described in detail elsewhere^16,17^ and within the eMethods in Supplement 1. The study was approved by the St Jude Children’s Research Hospital Institutional Review Board and each of the participating centers in CCSS, with all participants providing written informed consent. The study followed the Strengthening the Reporting of Observational Studies in Epidemiology (STROBE) reporting guideline, ensuring comprehensive and transparent reporting of the study design, data collection, and statistical analysis.

### Ascertainment of Late-Onset CCM

In SJLIFE, CCM was clinically assessed based on ejection fraction and pharmacologic treatment. In CCSS, CCM was determined through self-reported diagnoses and pharmacologic treatment. Severity of CCM was classified using the National Cancer Institute’s Common Terminology Criteria for Adverse Events (CTCAE), version 4.03,^18,19^ with grades of 2 or greater considered as affected. Further details are provided in the eMethods in Supplement 1.

### Cancer Treatment Exposures

Information on exposures to anthracyclines and radiation therapy was abstracted from participants’ medical records by SJLIFE and CCSS. Anthracycline doses were converted to doxorubicin equivalents as described earlier.^20^ Mean radiation dose to the heart was estimated using previously established radiation dosimetry methods.^21^

### Genetic Data

The whole-genome sequencing paired-end reads were generated using HiSeq XTen and/or NovaSeq (Illumina, Inc) for SJLIFE and a subset of CCSS survivors diagnosed between 1987 and 1999.^22^ Sequencing reads were mapped to the Genome Reference Consortium Human Build 38 reference genome using Burrows-Wheeler Aligner, version 0.7.12,^23^ and variants were called using Genome Analysis Toolkit, version 3.4.0^24,25^ following its recommended best practices. Samples with excess missingness (≥5%), cryptic relatedness (identity-by-descent proportion >0.20), and excess heterozygosity (>3 SDs from the mean value) were excluded. Variants with minimum genotype quality score less than 20, minimum mean read depth less than 10, Hardy-Weinberg equilibrium *P* < .001, and more than 10% missingness were also excluded. For CCSS participants diagnosed with cancer between 1970 and 1986, genotyping was performed using a HumanOmni5Exome (Illumina, Inc) array followed by imputation.^26^ Prior to imputation, samples with 8% or more missingness, per-sample heterozygosity of less than 0.11 or greater than 0.16, sex discordance, and first-degree relatives were excluded, along with variants with Hardy-Weinberg equilibrium *P* < .001, call rate of 97.0% or less, and an ambiguous strand (A/T or C/G).^27,28^ In both SJLIFE and CCSS, principal component analysis inferred genetic ancestry of participants on the basis of an independent set of common autosomal genetic variants and the reference 1000 Genomes Project populations.^29^

### Statistical Analysis

Associations of *TTN* (rs3829746-C) and *BAG3* (rs2234962-C) with late-onset CCM risk were examined separately in European ancestry survivors from the SJLIFE and CCSS cohorts, as well as in a combined European ancestry sample from both cohorts. Additionally, we examined these SNVs in SJLIFE African ancestry survivors. These analyses were conducted using logistic regression, adjusting for age at cancer diagnosis, age at last contact, sex, cumulative anthracycline dose, mean heart radiation dose, treatment era, and the top 10 principal components derived from the genotype data. The analyses were also stratified by sex, risk groups defined by the International Late Effects of Childhood Cancer Guideline Harmonization Group (IGHG), and cancer treatment exposures (eMethods in Supplement 1). Cohort-specific results were combined using a fixed-effects model in METAL.^30^

In the SJLIFE cohort, we further characterized associations of rs3829746-C and rs2234962-C with 7 echocardiography parameters (as continuous variables) of cardiac structure and function (left ventricular [LV] ejection fraction, LV end-diastolic volume, LV end-systolic volume, LV stroke volume, LV mass index, LV relative wall thickness, and global longitudinal peak strain) measured at SJLIFE baseline. Linear regression models assessed the association of each of the 2 SNVs with each parameter, adjusting for the same covariates used in the aforementioned logistic regression analysis.

The analyses of rare PAVs (minor allele frequency <1.0 × 10^−4^ per ancestry-matched population data from Genome Aggregation Database, version 4.1^31^) in *TTN* and *BAG3* in association with late-onset CCM risk were performed in SJLIFE and CCSS survivors separately using Fisher exact tests and in the combined sample using the Cochran-Mantel-Haenszel 2-by-2 test (eMethods in Supplement 1). These analyses were stratified by ancestry. In addition to *TTN* and *BAG3*, we examined carrier status of rare PAVs in 7 other genes (*DSP*, *LMNA*, *MYH7*, *SCN5A*, *TCAP*, *TNNC1*, and *TNNT2*), which have been previously associated with familial DCM in the general population.^3^

Further analyses were conducted with additional adjustments for follow-up duration; coronary artery disease (CTCAE grade ≥2); and cardiovascular risk factors such as diabetes (CTCAE grade ≥2), hypertension (CTCAE grade ≥2), dyslipidemia (CTCAE grade ≥2), and obesity (body mass index ≥30) that occurred prior to CCM. Individuals with missing information on CCM status or adjustment covariates were excluded from the analyses. The Bonferroni correction method was used to adjust for multiple testing in both analyses of common and rare variants. A 2-sided *P* < .05 was considered statistically significant. The statistical analyses were performed between January 4, 2023, and March 6, 2025, using R, version 4.1 (R Foundation for Statistical Computing).

## Results

Table 1 presents the clinical characteristics of survivors in the SJLIFE and CCSS cohorts. The prevalence of late-onset CCM was higher in SJLIFE (European ancestry survivors: 205 of 1605 [12.8%]; median [IQR] age at last contact, 43.9 [37.4-51.1] years; 84 female [41.0%] and 121 male [59.0%]; African ancestry survivors: 37 of 238 [15.5%]; median [IQR] age at last contact, 42.3 [35.2-47.6] years; 9 female [24.3%] and 28 male [75.7%]) compared with CCSS (248 of 4577 survivors [5.4%]; median [IQR] age at last contact, 42.7 [35.7-49.8] years; 152 female [61.3%] and 96 male [38.7%]). The median time from cancer diagnosis or cancer therapy exposures to late-onset CCM was 28.5 years (IQR, 22.1-34.8 years) among SJLIFE European ancestry survivors, 27.6 years (IQR, 22.5-32.0 years) among SJLIFE African ancestry survivors, and 21.8 years (IQR, 13.2-28.5 years) among CCSS survivors.

**Table 1.** Characteristics of Long-Term Survivors of Childhood Cancer Included in the Study

Characteristic	SJLIFE European ancestry		CCSS European ancestry		SJLIFE African ancestry	
	With late CCM (n = 205)	Without late CCM (n = 1400)	With late CCM (n = 248)	Without late CCM (n = 4329)	With late CCM (n = 37)	Without late CCM (n = 201)
Age at primary cancer diagnosis, median (IQR), y	10.8 (4.9 to 15.0)	7.1 (3.3 to 13.2)	11.7 (5.6 to 15.9)	8.5 (3.7 to 14.0)	9.4 (5.7 to 14.6)	8.6 (3.7 to 13.5)
Age at last contact, median (IQR), y	43.9 (37.4 to 51.1)	38.9 (33.2 to 46.4)	42.7 (35.7 to 49.8)	37.5 (31.2 to 44.8)	42.3 (35.2 to 47.6)	37.2 (31.4 to 44.4)
Time from cancer diagnosis to CCM, median (IQR), y	28.5 (22.1 to 34.8)	NA	21.8 (13.2 to 28.5)	NA	27.6 (22.5 to 32.0)	NA
Echocardiographic parameters, median (IQR)						
LV ejection fraction, %	46.6 (43.4 to 48.8)	56.7 (54.5 to 59.4)	NA	NA	44.6 (40.0 to 48.0)	57.0 (54.7 to 60.0)
LV end-diastolic volume, mL	107.5 (88.8 to 126.2)	100.9 (83.0 to 120.1)	NA	NA	113.0 (100.6 to 127.9)	95.5 (80.8 to 113.3)
LV end-systolic volume, mL	51.5 (41.9 to 66.2)	42.1 (34.0 to 51.5)	NA	NA	54.3 (45.4 to 66.1)	39.6 (31.2 to 46.2)
LV stroke volume, mL	54.7 (43.3 to 65.8)	57.6 (48.0 to 69.8)	NA	NA	55.7 (46.6 to 66.2)	54.9 (45.8 to 67.7)
LV mass index^a^	73.2 (59.4 to 86.8)	67.1 (56.4 to 81.0)	NA	NA	71.6 (63.1 to 93.2)	66.6 (57.5 to 80.5)
Global longitudinal peak strain, %	−17.4 (−19.1 to −15.0)	−19.5 (−21.1 to −17.8)	NA	NA	−15.7 (−18.1 to −12.8)	−18.9 (−20.4 to −17.4)
LV relative wall thickness	0.4 (0.3 to 0.5)	0.4 (0.3 to 0.4)	NA	NA	0.4 (0.4 to 0.4)	0.4 (0.4 to 0.5)
Sex, No. (%)						
Female	84 (41.0)	663 (47.4)	152 (61.3)	2208 (51.0)	9 (24.3)	107 (53.2)
Male	121 (59.0)	737 (52.6)	96 (38.7)	2121 (49.0)	28 (75.7)	94 (46.8)
Cumulative anthracycline dose, No. (%)						
0 mg/m^2^	51 (24.9)	331 (23.6)	72 (29.0)	2043 (47.2)	10 (27.0)	53 (26.4)
1-250 mg/m^2^	90 (43.9)	854 (61.0)	57 (23.0)	1420 (32.7)	13 (35.1)	123 (61.2)
>250 mg/m^2^	64 (31.2)	215 (15.4)	119 (48.0)	866 (20.0)	14 (37.8)	25 (12.4)
Mean heart radiation dose, No. (%)						
0 Gy	62 (30.2)	521 (37.2)	62 (25.0)	1109 (25.6)	9 (24.3)	76 (37.8)
1-25 Gy	116 (56.6)	827 (59.1)	133 (53.6)	2937 (67.8)	20 (54.1)	119 (59.2)
>25 Gy	27 (13.2)	52 (3.7)	53 (21.4)	283 (6.5)	8 (21.6)	6 (3.0)
Primary cancer diagnosis era, No. (%)						
1958-1968	7 (3.4)	26 (1.9)	0	0	2 (5.4)	4 (2.0)
1969-1978	52 (25.4)	243 (17.4)	103 (41.5)	1241 (28.7)	6 (16.2)	24 (11.9)
1979-1988	77 (37.6)	485 (34.6)	115 (46.4)	1879 (43.4)	15 (40.5)	58 (28.9)
1989-1998	62 (30.2)	526 (37.6)	28 (11.3)	1106 (25.5)	12 (32.4)	95 (47.3)
1999-2008	7 (3.4)	119 (8.5)	2 (0.8)	103 (2.4)	2 (5.4)	20 (10.0)
Comorbidities, No. (%)^b^						
Coronary artery disease	22 (10.7)	75 (5.4)	22 (8.9)	111 (2.6)	7 (18.9)	5 (2.5)
Diabetes	30 (14.6)	185 (13.2)	10 (4.0)	208 (4.8)	9 (24.3)	26 (12.9)
Hypertension	93 (45.4)	451 (32.2)	46 (18.5)	676 (15.6)	22 (59.5)	74 (36.8)
Dyslipidemia	78 (38.0)	314 (22.4)	32 (12.9)	630 (14.6)	10 (27.0)	23 (11.4)
Obesity	128 (62.4)	999 (71.4)	34 (13.7)	1267 (29.3)	16 (43.2)	153 (76.1)

Abbreviations: CCM, cancer therapy–related cardiomyopathy; CCSS, Childhood Cancer Survivor Study; LV, left ventricular; NA, not applicable; SJLIFE, St Jude Lifetime Cohort.

^a^ Calculated as LV mass in grams divided by height in meters squared.

^b^ Prevalence of comorbidities prior to CCM.

### Common SNVs in *TTN* and *BAG3* and Their Association With CCM Risk

In SJLIFE European ancestry participants, minor alleles of both common missense SNVs in *TTN* (rs3829746-C: odds ratio [OR], 0.73; 95% CI, 0.55-0.97; *P* = .03) and *BAG3* (rs2234962-C: OR, 0.73; 95% CI, 0.55-0.96; *P* = .03) were significantly associated with a reduced risk of late-onset CCM (Table 2). While the direction of associations was consistent, with somewhat reduced amounts, in CCSS European ancestry survivors, they did not achieve statistical significance for rs3829746-C (OR, 0.88; 95% CI, 0.69-1.11; *P* = .28) and rs2234962-C (OR, 0.83; 95% CI, 0.65-1.07; *P* = .15). When data from both cohorts of European ancestry survivors were combined using a fixed-effects meta-analytic approach, both rs3829746-C (OR, 0.81; 95% CI, 0.68-0.97; *P* = .03) and rs2234962-C (OR, 0.79; 95% CI, 0.65-0.95; *P* = .01) were significantly associated with a decreased risk of late-onset CCM.

**Table 2.** Association of *TTN* (rs3829746) and *BAG3* (rs2234962) With CCM Risk in Long-Term Survivors of Childhood Cancer From the SJLIFE and CCSS Cohorts

Cohort	No. of survivors	*TTN* (rs3829746-C)					*BAG3* (rs2234962-C)				
		Survivors, No. (%)		EAF	OR (95% CI)^a^	*P* value	Survivors, No. (%)		EAF	OR (95% CI)^a^	*P* value
		With variant and CCM	With variant and without CCM				With variant and CCM	With variant and without CCM			
SJLIFE European ancestry	1605	67 (32.7)	560 (40.1)	0.22	0.73 (0.55-0.97)	.03	64 (31.2)	537 (38.4)	0.21	0.73 (0.55-0.96)	.03
CCSS European ancestry	4577	92 (37.1)	1730 (40.0)	0.22	0.88 (0.69-1.11)	.28	83 (33.5)	1601 (37.0)	0.20	0.83 (0.65-1.07)	.15
SJLIFE and CCSS European ancestry	6182	159 (35.1)	2290 (40.0)	0.22	0.81 (0.68-0.97)	.03	147 (32.5)	2138 (37.4)	0.21	0.79 (0.65-0.95)	.01
SJLIFE African ancestry	238	30 (81.18)	158 (78.6)	0.45	1.67 (0.86-3.23)	.13	2 (5.4)	14 (7.0)	0.03	0.25 (0.03-1.99)	.19

Abbreviations: CCM, cancer therapy–related cardiomyopathy; CCSS, Childhood Cancer Survivor Study; EAF, effect allele frequency; OR, odds ratio; SJLIFE, St Jude Lifetime Cohort.

^a^ Analyses were adjusted for sex, age at diagnosis, age at last contact, cumulative anthracycline dose, mean heart radiation dose, treatment era, and top 10 principal components based on the genotype data.

In addition to rs3829746 and rs2234962, we evaluated 31 common missense SNVs within *TTN* and 1 SNV within *BAG3*. Of these, 12 SNVs within *TTN* showed nominally significant (ie, *P* = .02 to .05) associations with late-onset CCM risk in the combined sample of European ancestry survivors, as shown in eTable 1 in Supplement 1. However, none remained significant after Bonferroni correction for multiple testing (.05 / 32; *P* = .002). Minor alleles of 11 SNVs were nominally associated with a decreased risk of late-onset CCM, while 1 SNV was associated with an increased risk. In SJLIFE African ancestry survivors, minor allele T of rs3858340 within *BAG3* was associated with an increased late-onset CCM risk, showing a more pronounced association than rs2234962-C (OR, 2.70; 95% CI, 1.21-6.05; *P* = .02) (eTable 1 in Supplement 1). However, this association was not observed in European ancestry survivors from SJLIFE or CCSS, and it should be further examined in independent African ancestry survivors. Similar results were obtained in analyses further adjusted for follow-up duration, coronary artery disease, and cardiovascular risk factors (eTable 2 in Supplement 1).

### Stratified Analyses by Sex, Cancer Treatment Exposures, and IGHG Risk Groups

In the combined sample of European ancestry survivors from SJLIFE and CCSS, *TTN* (rs3829746-C) showed a consistent, but nonsignificant, increased late-onset CCM risk in female (n = 3105) and male (n = 3075) participants and IGHG high-risk (n = 1723) and moderate-risk (n = 1213) groups (eTable 3 in Supplement 1). However, an association was observed between rs3829746-C and a reduced risk of late-onset CCM among low-risk survivors (n = 2245), with a greater odds compared with that observed in the overall group of survivors (OR, 0.48; 95% CI, 0.25-0.95; *P* = .03). Among anthracycline-treated (with or without heart radiotherapy) survivors (n = 3683), rs3829746-C was significantly associated with reduced late-onset CCM risk (OR, 0.79; 95% CI, 0.64-0.97; *P* = .03) but not in those exposed to heart radiotherapy with or without anthracyclines (n = 4426).

For *BAG3* (rs2234962-C), a significant association with reduced CCM risk was observed in male survivors (n = 3074; OR, 0.69; 95% CI, 0.53-0.91; *P* = .007), while no significant result was found in female survivors (n = 3103). In the high-risk group (n = 1722), rs2234962-C was significantly associated with reduced CCM risk (OR, 0.74; 95% CI, 0.57-0.96; *P* = .02) but not in the moderate-risk (n = 1213) or low-risk (n = 2242) groups. A significant association was found in survivors treated with anthracyclines with or without heart radiotherapy (n = 3683; OR, 0.78; 95% CI, 0.63-0.97; *P* = .03) and those exposed to heart radiotherapy with or without anthracyclines (n = 4423; OR, 0.79; 95% CI, 0.63-0.97; *P* = .03).

### Associations of *TTN* (rs3829746-C) and *BAG3* (rs2234962-C) Variants With Cardiac Structure and Function

In SJLIFE European ancestry survivors, both variants were significantly associated with lower LV end-systolic volume (rs3829746-C: β [SE], −1.90 [0.65]; *P* = .003; rs2234962-C: β [SE], −2.68 [0.64]; *P* = .003) and global longitudinal peak strain (rs3829746-C: β [SE], −0.31 [0.13]; *P* = .02; rs2234962-C: β [SE], −0.30 [0.12]; *P* = .02) (eTable 4 in Supplement 1). The rs2234962-C variant was also associated with lower LV end-diastolic volume (β [SE], −3.38 [1.14]; *P* = .003). Both variants were associated with increased LV ejection fraction (rs3829746-C: β [SE], 0.62 [0.27]; *P* = .02; rs2234962-C: β [SE], 0.86 [0.27]; *P* = .001). In SJLIFE African ancestry survivors, rs3829746-C was not associated with any parameter, but rs2234962-C was associated with higher LV relative wall thickness in male survivors (β [SE], 0.09 [0.04]; *P* = .02) and to LV end-diastolic volume (β [SE], 17.79 [8.76]; *P* = .05) and stroke volume (β [SE], 11.31 [4.98]; *P* = .03) in female survivors. Consistent results were observed when analyzing these parameters from both baseline and follow-up SJLIFE visits using multivariable linear mixed-effects models that accounted for individual-specific random intercepts (eTable 5 in Supplement 1).

### Rare PAVs

Rare PAVs identified in European and African ancestry survivors are listed in eTables 6 and 7 in Supplement 1. Among European ancestry survivors, the prevalence of rare PAVs in *TTN* exons (percent sliced in [PSI] >0.82) was 0.18% (n = 3) in SJLIFE and 0.13% (n = 6) in CCSS. For *TTN* exons in the A-band region, the prevalence was 0.06% (n = 1) and 0% (n = 0), respectively (eTable 8 in Supplement 1). The prevalence of rare PAVs in *BAG3* was 0.50% (n = 8) in SJLIFE and 0.26% (n = 12) in CCSS. Across 9 genes, including *TTN* and *BAG3*, rare PAV prevalence was 2.61% (n = 42) in SJLIFE and 1.36% (n = 61) in CCSS. However, no significant association was found between late-onset CCM risk and PAV carrier status in *TTN* exons with PSI greater than 0.82, *TTN* exons with PSI greater than 0.82, in the A-band region, or in *BAG3* (eTable 8 in Supplement 1) or in African ancestry survivors (eTable 9 in Supplement 1). Expanding the analysis to include 7 additional genes associated with familial DCM revealed no significant associations with late-onset CCM risk in either European or African ancestry survivors. Consistent results were obtained when analyses were further adjusted for follow-up duration, coronary artery disease, and cardiovascular risk factors (eTable 10 in Supplement 1).

## Discussion

This cohort study found that common missense variants in *TTN* and *BAG3*, previously associated with a lower risk of sporadic DCM in the general population,^7,13,32^ were also associated with a reduced risk of late-onset CCM among childhood cancer survivors. Conversely, rare PAVs in these and other genes related to a higher risk of familial DCM did not show an association with late-onset CCM risk in survivors. These results suggest that akin to sporadic DCM in the general population, the risk of late-onset CCM in childhood cancer survivors may be more influenced by common variants within *TTN* and *BAG3* than rare ones.

Garcia-Pavia et al^3^ found that 16 of 213 adult and pediatric cancer survivors with CCM (7.5%) across 3 cohorts carried rare PAVs in familial DCM genes, particularly *TTN*. This prevalence was significantly (*P* < .001) higher compared with patients with breast and lung cancer from the Cancer Genome Project, healthy volunteers, and individuals from the Genome Aggregation Database. Among European ancestry individuals, the prevalence of rare PAVs in *TTN* was 7.1%. In contrast, our study of 238 participants with late-onset CCM among 5-year European ancestry survivors of childhood cancer found no rare PAVs in *TTN*. The prevalence among survivors without late-onset CCM was 0.21%, slightly lower than 0.70% in the large cohort of healthy volunteers reported by Garcia-Pavia et al. Among 37 African ancestry survivors with late-onset CCM, only 1 carried a rare *TTN* PAV. Several factors may explain this discrepancy. First, the time between cancer treatment and CCM diagnosis in Garcia-Pavia et al was short, particularly for the 42 childhood cancer survivors (0.3 years), suggesting that early-onset CCM may have been due to cancer therapies. In contrast, our study had a much longer duration from cancer diagnosis to CCM (28.5 years in SJLIFE and 21.8 years in CCSS), indicating late-onset CCM. Second, only 42 of the 213 patients with CCM (19.7%) in Garcia-Pavia et al were childhood cancer survivors, and this group had the lowest prevalence of rare PAVs (4.9%) compared with survivors of various adult cancers (eg, breast, hematologic, other solid tumors) in Spain and the UK (10.0%) and of breast cancer in the US (5.5%). Third, while Garcia-Pavia et al identified 16 rare PAVs in *TTN*, we found 6 rare PAVs in *TTN* exons with PSI greater than 0.82, none of which were reported in their study, suggesting differences that require further investigation. These results suggest that while cardiotoxic cancer therapies may unmask the harmful cardiac effects of rare PAVs in *TTN* and other DCM genes, leading to early-onset CCM, these variants may not significantly increase the risk of late-onset CCM, which typically develops years to decades after a childhood cancer diagnosis. This finding aligns with other population-based studies, which have suggested that rare variants may be more likely to be associated with early-onset diseases.^33,34^

We also identified common missense SNVs in *TTN* (rs3829746-C) and *BAG3* (rs2234962-C) that have been previously associated with a lower risk of sporadic DCM in the general population.^7,32^ These SNVs were also associated with a 19% reduced risk of late-onset CCM in childhood cancer survivors, with similar magnitudes of association. The association between *TTN* (rs3829746-C) and late-onset CCM risk was more pronounced among survivors in the IGHG-based low-risk group, who were exposed to lower doses of cardiotoxic therapies, with a 52% reduction in odds. In contrast, the higher doses of cardiotoxic treatments in the high- and moderate-risk groups may have diminished the SNV’s association with late-onset CCM in these groups. Conversely, *BAG3* (rs2234962-C) was not associated with CCM risk in the low-risk group but was associated with a 26% reduction in the high-risk group, where higher doses of cardiotoxic therapies might have slightly modified the SNV’s association. However, further research is needed to explore this observation.

### Limitations

Several limitations should be considered when interpreting our results. Our study focused on childhood cancer survivors who had lived at least 5 years after diagnosis and provided a blood sample for genotyping or sequencing. However, excluding survivors who died of cardiac causes before participating may have biased our genetic associations toward the null. Additionally, only a few echocardiographic parameters were collected in SJLIFE, with no data available from CCSS. A diagnosis of CCM was clinically assessed in SJLIFE but self-reported in CCSS, which may have contributed to a higher prevalence of CCM and potentially attenuated the associations between genetic variants in *TTN* and *BAG3* and CCM risk. Our analysis was also limited by low statistical power due to the low prevalence of both CCM and PAV carriers. Sufficient power was only achieved with a strong positive correlation between PAV carrier status and CCM risk, requiring at least a 5-fold increase in risk. While our study focused primarily on rare PAVs in 9 genes, given their established associations with the risks of DCM and early-onset CCM, future larger studies should also explore rare PAVs in other genes to better understand their potential role in the risk of late-onset CCM in childhood cancer survivors. The analysis of African ancestry survivors was constrained by small sample sizes, potentially underestimating associations between genetic variants and late-onset CCM risk.

## Conclusions

In this retrospective cohort study with prospective follow-up, rare PAVs within *TTN*, *BAG3*, and other familial DCM genes were not associated with the risk of late-onset CCM in survivors of childhood cancer. However, further research with larger sample sizes is needed to confirm this observation. In contrast, common missense variants in these genes were associated with a reduced risk of late-onset CCM, similar to their association with a lower risk of sporadic DCM in the general population.

## Notes

Supplement 1.
eMethods.

eReferences.
**eTable 1.** Common Missense SNVs Within *TTN* and *BAG3* and Their Associations With CCM Risk in Long-Term Survivors of Childhood Cancer From the St Jude Lifetime Cohort (SJLIFE) and Childhood Cancer Survivor Study (CCSS) and Through Meta-Analysis **eTable 2.** Common Missense SNVs Within *TTN* and *BAG3* and Their Associations With CCM Risk in Long-Term Survivors of Childhood Cancer From the St Jude Lifetime Cohort (SJLIFE) and Childhood Cancer Survivor Study (CCSS) and Through Meta-Analysis, With Additional Adjustment for Follow-Up Duration, Coronary Artery Disease, and Cardiovascular Risk Factors **eTable 3.** Association of *TTN* (rs3829746) and *BAG3* (rs2234962) With CCM Risk, Stratified by Sex, IGHG Risk Groups and Treatment Exposures, in Long-Term European Ancestry Survivors of Childhood Cancer From the St Jude Lifetime Cohort (SJLIFE) and Childhood Cancer Survivor Study (CCSS), and Through the Meta-Analysis **eTable 4.** Cross-Sectional Association of *TTN* (rs3829746-C) and *BAG3* (rs2234962-C) With Echocardiographic Parameters (Baseline) in the St Jude Lifetime Cohort (SJLIFE) **eTable 5.** Longitudinal Association of *TTN* (rs3829746-C) and *BAG3* (rs2234962-C) With Echocardiographic Parameters (Baseline and Follow-Up Visits) in the St Jude Lifetime Cohort (SJLIFE) **eTable 6.** Rare (Minor Allele Frequency [MAF] <1 × 10^-4^, per gnomAD v4.1 Non-Finnish European [NFE] Population) Protein-Altering Variants (PAV) Within Familial Dilated Cardiomyopathy Genes, Identified in Childhood Cancer Survivors of European Ancestry **eTable 7.** Rare (Minor Allele Frequency [MAF] <1 × 10^-4^, per gnomAD v4.1 African Population) Protein-Altering Variants (PAVs) Within Familial Dilated Cardiomyopathy Genes, Identified in Childhood Cancer Survivors of African Ancestry **eTable 8.** Association of Rare Protein-Altering Variants (PAVs) in Familial DCM-Related Genes From the General Population, With Late-Onset CCM Risk in Long-Term Childhood Cancer Survivors of European Ancestry From the St Jude Lifetime Cohort (SJLIFE) and Childhood Cancer Survivor Study (CCSS) **eTable 9.** Association of Rare Protein-Altering Variants (PAVs) Within Familial DCM-Related Genes From the General Population in Long-Term Survivors of Childhood Cancer From the St Jude Lifetime Cohort of African Ancestry **eTable 10.** Association Of Rare Protein-Altering Variants (PAVs) in Familial DCM-Related Genes From the General Population, With Late-Onset CCM Risk in Long-Term Childhood Cancer Survivors of European Ancestry from the St Jude Lifetime Cohort (SJLIFE) and Childhood Cancer Survivor Study (CCSS), With Additional Adjustment for Follow-Up Duration, Coronary Artery Disease (CAD), and Cardiovascular Risk Factors

Supplement 2.
Data Sharing Statement
