# Supplementary material for: TTN and BAG3 in Cancer Therapy–Related Cardiomyopathy Among Long-Term Survivors of Childhood Cancer
Source: JAMA Netw Open. 2025 Jun 20;8(6):e2515793. doi: 10.1001/jamanetworkopen.2025.15793 (PMC12181783; doi:10.1001/jamanetworkopen.2025.15793)
Supplement: Supplement 1. — eMethods. eReferences. eTable 1. Common Missense SNVs Within TTN and BAG3 and Their Associations With CCM Risk in Long-Term Survivors of Childhood Cancer From the St Jude Lifetime Cohort (SJLIFE) and Childhood Cancer Survivor Study (CCSS) and Through Meta-Analysis eTable 2. Common Missense SNVs Within TTN and BAG3 and Their Associations With CCM Risk in Long-Term Survivors of Childhood Cancer From the St Jude Lifetime Cohort (SJLIFE) and Childhood Cancer Survivor Study (CCSS) and Through Meta-Analysis, With Additional Adjustment for Follow-Up Duration, Coronary Artery Disease, and Cardiovascular Risk Factors eTable 3. Association of TTN (rs3829746) and BAG3 (rs2234962) With CCM Risk, Stratified by Sex, IGHG Risk Groups and Treatment Exposures, in Long-Term European Ancestry Survivors of Childhood Cancer From the St Jude Lifetime Cohort (SJLIFE) and Childhood Cancer Survivor Study (CCSS), and Through the Meta-Analysis eTable 4. Cross-Sectional Association of TTN (rs3829746-C) and BAG3 (rs2234962-C) With Echocardiographic Parameters (Baseline) in the St Jude Lifetime Cohort (SJLIFE) eTable 5. Longitudinal Association of TTN (rs3829746-C) and BAG3 (rs2234962-C) With Echocardiographic Parameters (Baseline and Follow-Up Visits) in the St Jude Lifetime Cohort (SJLIFE) eTable 6. Rare (Minor Allele Frequency [MAF] <1 × 10^-4^, per gnomAD v4.1 Non-Finnish European [NFE] Population) Protein-Altering Variants (PAV) Within Familial Dilated Cardiomyopathy Genes, Identified in Childhood Cancer Survivors of European Ancestry eTable 7. Rare (Minor Allele Frequency [MAF] <1 × 10^-4^, per gnomAD v4.1 African Population) Protein-Altering Variants (PAVs) Within Familial Dilated Cardiomyopathy Genes, Identified in Childhood Cancer Survivors of African Ancestry eTable 8. Association of Rare Protein-Altering Variants (PAVs) in Familial DCM-Related Genes From the General Population, With Late-Onset CCM Risk in Long-Term Childhood Cancer Survivors of European Ancestry From the St Jude Lifetime [file jamanetwopen-e2515793-s001.pdf]

Supplemental Online Content

Neupane A, Petrykey K, Li K, et al. *TTN* and *BAG3* in cancer therapy–related cardiomyopathy among long-term survivors of childhood cancer. *JAMA Netw Open*. 2025;8(6):e2515793. doi:10.1001/jamanetworkopen.2025.15793

eMethods.

eReferences.

eTable 1. Common Missense SNVs Within *TTN* and *BAG3* and Their Associations With CCM Risk in Long-Term Survivors of Childhood Cancer From the St Jude Lifetime Cohort (SJLIFE) and Childhood Cancer Survivor Study (CCSS) and Through Meta-Analysis

eTable 2. Common Missense SNVs Within *TTN* and *BAG3* and Their Associations With CCM Risk in Long-Term Survivors of Childhood Cancer From the St Jude Lifetime Cohort (SJLIFE) and Childhood Cancer Survivor Study (CCSS) and Through Meta-Analysis, With Additional Adjustment for Follow-Up Duration, Coronary Artery Disease, and Cardiovascular Risk Factors

eTable 3. Association of *TTN* (rs3829746) and *BAG3* (rs2234962) With CCM Risk, Stratified by Sex, IGHG Risk Groups and Treatment Exposures, in Long-Term European Ancestry Survivors of Childhood Cancer From the St Jude Lifetime Cohort (SJLIFE) and Childhood Cancer Survivor Study (CCSS), and Through the Meta-Analysis

eTable 4. Cross-Sectional Association of *TTN* (rs3829746-C) and *BAG3* (rs2234962-C) With Echocardiographic Parameters (Baseline) in the St Jude Lifetime Cohort (SJLIFE)

eTable 5. Longitudinal Association of *TTN* (rs3829746-C) and *BAG3* (rs2234962-C) With Echocardiographic Parameters (Baseline and Follow-Up Visits) in the St Jude Lifetime Cohort (SJLIFE)

eTable 6. Rare (Minor Allele Frequency [MAF] <1 × 10^{-4}, per gnomAD v4.1 Non-Finnish European [NFE] Population) Protein-Altering Variants (PAV) Within Familial Dilated Cardiomyopathy Genes, Identified in Childhood Cancer Survivors of European Ancestry

eTable 7. Rare (Minor Allele Frequency [MAF] <1 × 10^{-4}, per gnomAD v4.1 African Population) Protein-Altering Variants (PAVs) Within Familial Dilated Cardiomyopathy Genes, Identified in Childhood Cancer Survivors of African Ancestry

eTable 8. Association of Rare Protein-Altering Variants (PAVs) in Familial DCM-Related Genes From the General Population, With Late-Onset CCM Risk in Long-Term Childhood Cancer Survivors of European Ancestry From the St Jude Lifetime Cohort (SJLIFE) and Childhood Cancer Survivor Study (CCSS)

eTable 9. Association of Rare Protein-Altering Variants (PAVs) Within Familial DCM-Related Genes From the General Population in Long-Term Survivors of Childhood Cancer From the St Jude Lifetime Cohort of African Ancestry

© 2025 Neupane A et al. *JAMA Network Open*.

eTable 10. Association Of Rare Protein-Altering Variants (PAVs) in Familial DCM-Related Genes From the General Population, With Late-Onset CCM Risk in Long-Term Childhood Cancer Survivors of European Ancestry from the St Jude Lifetime Cohort (SJLIFE) and Childhood Cancer Survivor Study (CCSS), With Additional Adjustment for Follow-Up Duration, Coronary Artery Disease (CAD), and Cardiovascular Risk Factors

This supplemental material has been provided by the authors to give readers additional information about their work.

© 2025 Neupane A et al. *JAMA Network Open*.

eMethods

Study population

SJLIFE is a retrospectively constructed cohort study with a prospective clinical follow-up and ongoing enrollment of five-year survivors treated for childhood cancer at the St. Jude Children's Research Hospital (SJCRH) since 1962. To be eligible for this analysis, SJLIFE participants had to have whole genome sequencing (WGS) data and be exposed to either anthracyclines or chest-directed radiotherapy. A total of 1,605 survivors of European ancestry (EUR) and 238 survivors of African ancestry (AFR) met these criteria.

CCSS is also a retrospectively constructed cohort study with prospective follow-up surveys of 5-year survivors of childhood cancer treated at 31 institutions in the United States and Canada. Participants in CCSS include survivors diagnosed with cancer at <21 years of age between 1970 and 1999. To be eligible for this study, CCSS participants had to have genome-wide genotype, WGS or whole-exome sequencing data, be of European ancestry and be exposed to either anthracyclines or chest-directed radiotherapy. Due to the limited number of CCSS participants of African ancestry with available genome-wide genotype data, they were excluded from this analysis. A total of 4,577 CCSS survivors met these criteria. The Institutional Review Boards at SJCRH and each of the centers participating in the CCSS approved the study. All study participants provided written informed consent.

Ascertainment of CCM

In SJLIFE, CCM was clinically assessed based on ejection fraction (EF) and pharmacologic treatment. EF was quantified using two-dimensional Doppler

© 2025 Neupane A et al. *JAMA Network Open*.

echocardiography along with 3-dimensional imaging of left ventricular volumes, according to the American Society of Echocardiography guidelines[1]. CCM was classified as moderate (grade 2; resting EF <50-40% or 10-19% absolute drop from baseline), severe or disabling (grade 3; resting EF 39-20%; or >20% absolute drop from baseline; or medication initiated), life-threatening (grade 4; resting EF <20%; refractory or poorly controlled heart failure; intervention such as ventricular assist device, intravenous vasopressor support; or heart transplant indicated), or fatal (grade 5; death) based on modifications of the National Cancer Institute's Common Terminology Criteria for Adverse Events (CTCAE) version 4.03[2,3]. In CCSS, the presence of CCM was based on self-report of medical diagnosis ("Have you ever been told by a doctor or other health care provider that you have or have had heart failure") and pharmacologic treatment. Using CCSS' adaptation of the CTCAE v4.03[2,3], CCM was then graded as moderate (grade 2; self-reported heart failure not requiring medication), severe or disabling (grade 3; cardiomyopathy or heart failure requiring medication), life-threatening (grade 4; cardiac transplantation), or fatal (grade 5; death as a result of heart failure). In this analysis, survivors with CTCAE grade 2 or higher were considered affected and those with grade 0 as unaffected.

Statistical Analysis

Given that both rs2234962-C and rs3829746-C are missense variants, we also analyzed all other common (MAF>5%) missense SNVs within *TTN* and *BAG3* in EUR survivors from SJLIFE and CCSS cohorts, separately and through meta-analysis of cohort-specific results. Additionally, these analyses were stratified by sex and risk groups, defined by the International Late Effects of Childhood Cancer Guideline

© 2025 Neupane A et al. *JAMA Network Open*.

Harmonization Group[4]. Risk groups were categorized based on prior anthracycline and/or chest-directed radiation exposure. Survivors were classified into high-risk (cumulative anthracycline dose ≥250 mg/m^2, chest-directed radiotherapy ≥30 Gray [Gy], or a combination of cumulative anthracyclines ≥100 mg/m^2 and chest-directed radiotherapy ≥15 Gy), moderate-risk (100-249 mg/m^2 anthracyclines or chest-directed radiotherapy 15-29 Gy) and low-risk (1-99 mg/m^2 anthracyclines and chest-directed radiotherapy <15 Gy).

Variants were annotated using SnpEff[5] (version 5.1d), and those classified as missense, nonsense, frameshift, in-frame insertion or deletion variants were considered PAVs, following the same approach described by Garcia-Pavia et al[6]. Given that *TTN* truncating variants associated with DCM are predominantly found in exons that are highly expressed in LV tissues, particularly those encoding the A-band[7], we examined *TTN* PAVs in constitutively expressed exons (PSI>0.82) using data from the Genotype-Tissue Expression project[8]. We also explored the association of *TTN* PAVs in constitutively expressed exons (PSI>0.82) within the A-band region of the gene.

Principal component analysis

Analysis was performed separately in SJLIFE and CCSS. In order to identify ancestry of the SJLIFE or CCSS samples, we combined the genotype data of common single-nucleotide variants (SNVs) with 26 global populations from the 1000 Genomes Project and performed EIGENSTRAT-based Principal Component Analysis (PCA)[9] to obtain the first two principal components (PC1 and PC2), implemented in the PLINK 1.90b software. The mean PC1 and PC2 scores of the 1000 Genomes European populations

© 2025 Neupane A et al. *JAMA Network Open*.

(CEU- Utah Residents (CEPH) with Northern and Western European Ancestry; TSI - Toscani in Italia; FIN - Finnish in Finland; GBR - British in England and Scotland; and IBS-Iberian Population in Spain) were used as a reference and any SJLIFE/CCSS sample within three standard deviations from these along PC1 or PC2 were classified as a European. Similarly, the mean PC1 and PC2 scores of the 1000 Genomes African populations (YRI - Yoruba in Ibadan, Nigeria; LWK - Luhya in Webuye, Kenya; GWD - Gambian in Western Divisions in the Gambia; MSL - Mende in Sierra Leone; ESN - Esan in Nigeria; ASW - Americans of African Ancestry in SW USA; ACB - African Caribbeans in Barbados) were used as a reference and any SJLIFE/CCSS sample within three standard deviations from these along PC1 or PC2 were classified as a African.

© 2025 Neupane A et al. *JAMA Network Open*.

eReferences

1. Lang RM, Bierig M, Devereux RB, et al. Recommendations for chamber quantification: a report from the American Society of Echocardiography's Guidelines and Standards Committee and the Chamber Quantification Writing Group, developed in conjunction with the European Association of Echocardiography, a branch of the European Society of Cardiology. *J Am Soc Echocardiogr*. Dec 2005;18(12):1440-63. doi:10.1016/j.echo.2005.10.005
2. Hudson MM, Ehrhardt MJ, Bhakta N, et al. Approach for Classification and Severity Grading of Long-term and Late-Onset Health Events among Childhood Cancer Survivors in the St. Jude Lifetime Cohort. *Cancer Epidem Biomar*. May 2017;26(5):666-674. doi:10.1158/1055-9965.Epi-16-0812
3. Oeffinger KC, Mertens AC, Sklar CA, et al. Chronic health conditions in adult survivors of childhood cancer. *N Engl J Med*. Oct 12 2006;355(15):1572-82. doi:10.1056/NEJMsa060185
4. Ehrhardt MJ, Leerink JM, Mulder RL, et al. Systematic review and updated recommendations for cardiomyopathy surveillance for survivors of childhood, adolescent, and young adult cancer from the International Late Effects of Childhood Cancer Guideline Harmonization Group. *Lancet Oncol*. Mar 2023;24(3):e108-e120. doi:10.1016/S1470-2045(23)00012-8
5. Cingolani P, Platts A, Wang LL, et al. A program for annotating and predicting the effects of single nucleotide polymorphisms, SnpEff: SNPs in the genome of Drosophila melanogaster strain w(1118); iso-2; iso-3. *Fly*. Apr-Jun 2012;6(2):80-92. doi:10.4161/fly.19695
6. Garcia-Pavia P, Kim Y, Restrepo-Cordoba MA, et al. Genetic Variants Associated With Cancer Therapy-Induced Cardiomyopathy. *Circulation*. Jul 2 2019;140(1):31-41. doi:10.1161/Circulationaha.118.037934
7. Herman DS, Lam L, Taylor MRG, et al. Truncations of Titin Causing Dilated Cardiomyopathy. *New Engl J Med*. Feb 16 2012;366(7):619-628. doi:DOI 10.1056/NEJMoa1110186
8. Consortium GT, Laboratory DA, Coordinating Center -Analysis Working G, et al. Genetic effects on gene expression across human tissues. *Nature*. Oct 11 2017;550(7675):204-213. doi:10.1038/nature24277
9. Price AL, Patterson NJ, Plenge RM, Weinblatt ME, Shadick NA, Reich D. Principal components analysis corrects for stratification in genome-wide association studies. *Nat Genet*. Aug 2006;38(8):904-9. doi:10.1038/ng1847

© 2025 Neupane A et al. *JAMA Network Open*.

eTable 1. Common Missense SNVs Within *TTN* and *BAG3* and Their Associations With CCM Risk in Long-Term Survivors of Childhood Cancer From the St Jude Lifetime Cohort (SJLIFE) and Childhood Cancer Survivor Study (CCSS) and Through Meta-Analysis

Chr	Position (hg38)	rsID	Gene	EA	NEA	European ancestry								African ancestry			African and European ancestry meta-analysis	
						SJLIFE			CCSS			Meta-analysis		SJLIFE				
						EAF	OR (95% CI)	*P*	EAF	OR (95% CI)	*P*	OR (95% CI)	*P*	EAF	OR (95% CI)	*P*	OR (95% CI)	*P*
2	178571293	rs744426	*TTN*	A	G	0.14	0.71 (0.50-1.00)	.047	0.14	0.81 (0.61-1.08)	.16	0.77 (0.62-0.96)	.02	0.08	2.12 (0.68-6.56)	.19	0.80 (0.64-0.99)	.04
2	178566270	rs3731746	*TTN*	A	G	0.16	0.71 (0.51-0.98)	.03	0.17	0.84 (0.65-1.09)	.20	0.79 (0.64-0.96)	.02	0.33	1.37 (0.68-2.74)	.38	0.82 (0.67-0.10)	.05
2	178580212	rs2303838	*TTN*	T	C	0.19	0.72 (0.52-0.98)	.04	0.18	0.84 (0.65-1.09)	.20	0.79 (0.65-0.96)	.02	0.32	1.63 (0.82-3.25)	.17	0.84 (0.69-1.01)	.07
2	178586693	rs2042996	*TTN*	A	G	0.22	0.73 (0.55-0.97)	.03	0.22	0.86 (0.67-1.09)	.20	0.8 (0.67-0.96)	.02	0.48	1.70 (0.89-3.22)	.11	0.78 (0.66-0.94)	.01
2	178556967	rs9808377	*TTN*	G	A	0.22	0.73 (0.55-0.97)	.03	0.22	0.86 (0.68-1.09)	.22	0.81 (0.67-0.97)	.02	0.46	1.66 (0.85-3.21)	.13	0.79 (0.66-0.94)	.01
2	178532834	rs3829747	*TTN*	T	C	0.14	0.72 (0.51-1.01)	.06	0.15	0.83 (0.63-1.09)	.18	0.78 (0.63-0.97)	.03	0.08	2.23 (0.71-6.99)	.17	0.81 (0.66-1.00)	.05
2	178599800	rs1001238	*TTN*	C	T	0.22	0.72 (0.54-0.95)	.02	0.23	0.88 (0.69-1.11)	.28	0.81 (0.67-0.97)	.02	0.46	1.70 (0.89-3.26)	.11	0.79 (0.66-0.94)	.01
2	178592420	rs16866406	*TTN*	A	G	0.14	0.71 (0.51-1.00)	.05	0.15	0.83 (0.63-1.10)	.20	0.78 (0.63-0.97)	.03	0.08	2.11 (0.68-6.53)	.19	0.81 (0.66-1.00)	.05
2	178593864	rs2288569	*TTN*	T	C	0.14	0.72 (0.51-1.01)	.05	0.15	0.85 (0.64-1.12)	.25	0.79 (0.64-0.98)	.04	0.08	2.08 (0.67-6.40)	.20	0.82 (0.67-1.01)	.07
2	178693639	rs2042995	*TTN*	C	T	0.22	0.71 (0.54-0.95)	.02	0.23	0.9 (0.71-1.14)	.38	0.82 (0.68-0.98)	.03	0.44	1.83 (0.93-3.58)	.08	0.80 (0.67-0.95)	.01
2	178717810	rs2627043	*TTN*	T	G	0.20	0.75 (0.57-1.00)	.047	0.21	0.98 (0.77-1.24)	.85	0.88 (0.73-1.05)	.16	0.41	1.51 (0.80-2.84)	.20	0.92 (0.77-1.09)	.31
2	178541464	rs3731749	*TTN*	T	C	0.14	0.72 (0.51-1.01)	.06	0.15	0.83 (0.63-1.10)	.19	0.78 (0.63-0.97)	.03	0.08	2.12 (0.68-6.56)	.19	0.81 (0.66-1.00)	.05
2	178633315	rs6723526	*TTN*	C	T	0.12	1.34 (0.98-1.83)	.06	0.11	1.36 (1.02-1.80)	.04	1.35 (1.09-1.67)	.005	0.03	1.65 (0.24-11.29)	.61	1.35 (1.10-1.67)	.004
2	178718769	rs16866465	*TTN*	G	T	0.16	0.75 (0.55-1.03)	.08	0.16	0.93 (0.71-1.21)	.59	0.85 (0.70-1.04)	.12	0.17	0.88 (0.39-2.02)	.77	0.85 (0.70-1.04)	.11
2	178722403	rs12693166	*TTN*	G	C	0.16	0.75 (0.55-1.03)	.08	0.16	0.93 (0.72-1.22)	.62	0.85 (0.70-1.05)	.13	0.19	0.67 (0.28-1.59)	.36	0.84 (0.69-1.03)	.09
2	178714366	rs12693164	*TTN*	C	T	0.16	0.75 (0.55-1.03)	.08	0.16	0.94 (0.73-1.23)	.67	0.86 (0.70-1.05)	.15	0.17	0.62 (0.24-1.56)	.31	0.85 (0.70-1.03)	.10
2	178717600	rs13390491	*TTN*	T	C	0.15	0.76 (0.56-1.04)	.09	0.16	0.93 (0.72-1.22)	.61	0.86 (0.70-1.05)	.14	0.18	0.61 (0.24-1.54)	.30	0.85 (0.70-1.03)	.10
2	178795185	rs16866538	*TTN*	A	G	0.05	0.83 (0.49-1.42)	.50	0.05	0.71 (0.45-1.14)	.16	0.76 (0.54-1.08)	.13	0.18	0.62 (0.24-1.58)	.32	0.75 (0.54-1.03)	.08
2	178785681	rs35813871	*TTN*	A	G	0.24	0.82 (0.63-1.07)	.15	0.24	0.92 (0.73-1.15)	.45	0.88 (0.74-1.04)	.13	0.08	0.15 (0.03-0.79)	.03	0.86 (0.72-1.02)	.08
2	178780128	rs10497520	*TTN*	T	C	0.13	0.88 (0.63-1.23)	.45	0.13	0.82 (0.60-1.13)	.23	0.85 (0.68-1.07)	.17	0.45	0.93 (0.49-1.79)	.83	0.87 (0.70-1.08)	.22
2	178567458	rs12463674	*TTN*	G	A	0.30	1.14 (0.90-1.45)	.27	0.30	1.06 (0.87-1.29)	.59	1.09 (0.94-1.27)	.26	0.03	NA	NA	1.09 (0.94-1.27)	.26
2	178681132	rs36051007	*TTN*	T	C	0.30	1.13 (0.89-1.43)	.32	0.30	1.05 (0.86-1.29)	.62	1.08 (0.93-1.26)	.31	0.03	0.16 (0.01-2.67)	.20	1.08 (0.92-1.26)	.34
2	178689578	rs2244492	*TTN*	T	C	0.35	1.10 (0.87-1.38)	.42	0.36	1.01 (0.84-1.23)	.88	1.05 (0.91-1.22)	.52	0.39	1.30 (0.67-2.55)	.44	1.03 (0.90-1.20)	.65

© 2025 Neupane A et al. *JAMA Network Open*.

2	178710784	rs72648998	*TTN*	T	C	0.06	1.02 (0.65-1.60)	.92	0.06	0.85 (0.56-1.29)	.45	0.93 (0.68-1.26)	.62	0.01	2.47 (0.13-45.74)	.54	0.93 (0.69-1.27)	.67
2	178663651	rs2163008	*TTN*	T	C	0.05	0.92 (0.55-1.52)	.74	0.05	0.92 (0.59-1.42)	.69	0.92 (0.66-1.28)	.61	0.48	1.53 (0.77-3.06)	.22	1.01 (0.75-1.36)	.95
2	178747656	rs72648907	*TTN*	T	C	0.06	1.08 (0.71-1.65)	.70	0.05	0.80 (0.49-1.31)	.37	0.95 (0.69-1.31)	.77	0.01	38.45 (2.58-57.4)	.008	1.00 (0.73-1.38)	.98
2	178759031	rs2291310	*TTN*	C	T	0.07	0.95 (0.62-1.45)	.80	0.06	1.05 (0.73-1.52)	.77	1.01 (0.76-1.33)	.96	0.14	0.70 (0.28-1.71)	.43	0.98 (0.75-1.27)	.85
2	178764734	rs2291311	*TTN*	C	T	0.07	0.95 (0.62-1.45)	.80	0.06	1.05 (0.73-1.52)	.77	1.01 (0.76-1.33)	.96	0.15	0.88 (0.38-2.04)	.77	1.00 (0.76-1.30)	.97
2	178756224	rs7585334	*TTN*	C	T	0.07	0.95 (0.62-1.45)	.80	0.06	1.05 (0.73-1.52)	.78	1.01 (0.76-1.33)	.96	0.15	0.89 (0.39-2.06)	.79	1.00 (0.76-1.30)	.97
2	178741811	rs2627037	*TTN*	A	G	0.07	0.89 (0.58-1.37)	.61	0.07	1.05 (0.73-1.50)	.80	0.98 (0.75-1.29)	.90	0.44	1.43 (0.72-2.82)	.31	1.03 (0.80-1.33)	.80
2	178751160	rs922984	*TTN*	T	C	0.07	0.93 (0.61-1.43)	.74	0.07	1.03 (0.71-1.49)	.87	0.99 (0.75-1.30)	.93	0.41	1.51 (0.75-3.06)	.25	1.05 (0.81-1.35)	.74
10	119676774	rs3858340	*BAG3*	T	C	0.09	1.18 (0.82-1.69)	.37	0.09	0.98 (0.69-1.38)	.90	1.07 (0.84-1.37)	.59	0.16	2.70 (1.21-6.05)	.02	1.16 (0.92-1.47)	.22

CCM, cancer therapy-induced cardiomyopathy; EA, effect allele; NEA, non-effect allele; EAF, effect allele frequency; OR, odds ratio; CI, confidence interval

Analyses were adjusted for sex, age at diagnosis, age at last contact, cumulative anthracycline dose, average heart radiation dose, treatment era and top ten principal components based on the genotype data

© 2025 Neupane A et al. *JAMA Network Open*.

eTable 2. Common Missense SNVs Within *TTN* and *BAG3* and Their Associations With CCM Risk in Long-Term Survivors of Childhood Cancer From the St Jude Lifetime Cohort (SJLIFE) and Childhood Cancer Survivor Study (CCSS) and Through Meta-Analysis, With Additional Adjustment for Follow-Up Duration, Coronary Artery Disease, and Cardiovascular Risk Factors (CVRFs: hypertension, diabetes, dyslipidemia and obesity)

rsID	European ancestry						African ancestry	
	SJLIFE		CCSS		Meta-analysis		SJLIFE	
	OR (95% CI)	*P*	OR (95% CI)	*P*	OR (95% CI)	*P*	OR (95% CI)	*P*
rs3829746	0.73 (0.55-0.98)	.03	0.88 (0.69-1.11)	.30	0.82 (0.68-0.98)	.03	1.68 (0.75-3.77)	.20
rs744426	0.72 (0.51–1.02)	.06	0.81 (0.61–1.09)	.16	0.77 (0.62–0.96)	.02	1.20 (0.33–4.43)	.78
rs3731746	0.72 (0.52–0.99)	.04	0.84 (0.64–1.09)	.19	0.79 (0.64–0.97)	.02	1.48 (0.63–3.46)	.37
rs2303838	0.72 (0.52–0.99)	.04	0.84 (0.65–1.10)	.20	0.79 (0.65–0.97)	.02	1.82 (0.79–4.18)	.16
rs2042996	0.73 (0.55–0.98)	.04	0.86 (0.67–1.09)	.21	0.80 (0.67–0.97)	.02	1.59 (0.75–3.38)	.23
rs9808377	0.73 (0.55–0.97)	.03	0.86 (0.68–1.10)	.23	0.81 (0.67–0.97)	.02	1.67 (0.75–3.75)	.21
rs3829747	0.73 (0.52–1.03)	.07	0.82 (0.62–1.09)	.18	0.79 (0.63–0.98)	.03	1.73 (0.48–6.31)	.40
rs1001238	0.72 (0.54–0.96)	.02	0.88 (0.69–1.12)	.30	0.81 (0.68–0.97)	.03	1.71 (0.79–3.72)	.17
rs16866406	0.73 (0.52–1.02)	.07	0.83 (0.63–1.10)	.19	0.79 (0.63–0.98)	.03	1.20 (0.33–4.43)	.78
rs2288569	0.73 (0.52–1.03)	.07	0.85 (0.64–1.12)	.24	0.80 (0.64–0.99)	.04	1.18 (0.32–4.34)	.80
rs2042995	0.71 (0.53–0.95)	.02	0.89 (0.70–1.13)	.34	0.81 (0.68–0.98)	.03	1.71 (0.75–3.89)	.20
rs2627043	0.75 (0.56–1.00)	.05	0.98 (0.77–1.25)	.88	0.88 (0.73–1.06)	.17	1.34 (0.63–2.85)	.45
rs3731749	0.73 (0.52–1.03)	.08	0.83 (0.62–1.09)	.18	0.79 (0.63–0.98)	.03	1.20 (0.33–4.43)	.78
rs6723526	1.34 (0.98–1.84)	.07	1.38 (1.03–1.84)	.03	1.36 (1.10–1.68)	.005	1.31 (0.14–12.04)	.81
rs16866465	0.76 (0.55–1.04)	.09	0.93 (0.72–1.22)	.62	0.86 (0.70–1.05)	.14	0.77 (0.28–2.17)	.62
rs12693166	0.76 (0.55–1.04)	.09	0.94 (0.72–1.23)	.64	0.86 (0.70–1.06)	.15	0.54 (0.18–1.58)	.26
rs12693164	0.76 (0.55–1.05)	.09	0.95 (0.73–1.24)	.71	0.87 (0.71–1.06)	.17	0.44 (0.13–1.45)	.18
rs13390491	0.77 (0.56–1.06)	.11	0.94 (0.72–1.22)	.63	0.87 (0.71–1.06)	.17	0.44 (0.13–1.44)	.17
rs16866538	0.85 (0.50–1.46)	.56	0.73 (0.46–1.17)	.19	0.78 (0.55–1.11)	.17	0.49 (0.15–1.58)	.23
rs35813871	0.84 (0.64–1.10)	.20	0.89 (0.71–1.12)	.32	0.87 (0.73–1.03)	.11	0.12 (0.02–0.80)	.03
rs10497520	0.89 (0.64–1.25)	.49	0.84 (0.61–1.15)	.28	0.86 (0.68–1.09)	.21	0.89 (0.42–1.86)	.75
rs12463674	1.13 (0.89–1.43)	.34	1.08 (0.88–1.32)	.46	1.10 (0.94–1.28)	.23	NA	NA
rs36051007	1.11 (0.87–1.41)	.40	1.08 (0.88–1.32)	.48	1.09 (0.93–1.27)	.28	0.44 (0.02–8.34)	.58
rs2244492	1.08 (0.85–1.36)	.55	1.04 (0.85–1.26)	.71	1.05 (0.91–1.22)	.50	1.51 (0.67–3.43)	.32
rs72648998	1.05 (0.66–1.67)	.83	0.88 (0.58–1.33)	.54	0.95 (0.70–1.30)	.76	1.16 (0.01–160.34)	.95
rs2163008	0.89 (0.53–1.50)	.66	0.91 (0.59–1.42)	.68	0.90 (0.64–1.27)	.55	1.54 (0.67–3.51)	.31
rs72648907	1.08 (0.70–1.65)	.73	0.75 (0.46–1.24)	.26	0.93 (0.67–1.28)	.64	72.46 (1.26–4174.65)	.04
rs2291310	0.94 (0.61–1.45)	.78	1.06 (0.73–1.54)	.76	1.01 (0.76–1.34)	.96	0.69 (0.22–2.15)	.53
rs2291311	0.94 (0.61–1.45)	.78	1.06 (0.73–1.54)	.76	1.01 (0.76–1.34)	.96	0.88 (0.30–2.54)	.81
rs7585334	0.94 (0.61–1.45)	.78	1.06 (0.73–1.53)	.76	1.01 (0.76–1.33)	.96	0.88 (0.31–2.55)	.81
rs2627037	0.89 (0.57–1.36)	.58	1.06 (0.74–1.52)	.76	0.98 (0.75–1.30)	.90	1.38 (0.63–3.03)	.42
rs922984	0.93 (0.60–1.43)	.73	1.04 (0.71–1.50)	.86	0.99 (0.75–1.31)	.93	1.55 (0.69–3.48)	.29
rs2234962	0.73 (0.55-0.97)	.03	0.82 (0.64-1.06)	.13	0.78 (0.65-0.94)	.01	0.45 (0.05-3.98)	.47
rs3858340	1.15 (0.80–1.66)	.46	0.97 (0.68–1.37)	.85	1.05 (0.82–1.35)	.71	3.65 (1.41–9.41)	.01

CCM, cancer therapy-induced cardiomyopathy; OR, odds ratio; CI, confidence interval

© 2025 Neupane A et al. *JAMA Network Open*.

eTable 3. Association of *TTN* (rs3829746) and *BAG3* (rs2234962) With CCM Risk, Stratified by Sex, IGHG Risk Groups and Treatment Exposures, in Long-Term European Ancestry Survivors of Childhood Cancer From the St Jude Lifetime Cohort (SJLIFE) and Childhood Cancer Survivor Study (CCSS), and Through the Meta-Analysis

Variants	Strata	SJLIFE			CCSS			Meta-analysis	
		Total N (N with CCM)	OR (95% CI)	*P*	Total N (N with CCM)	OR (95% CI)	*P*	OR (95% CI)	*P*
TTN (rs3829746)	All	1603 (205)	0.73 (0.55-0.97)	.03	4577 (248)	0.88 (0.69-1.11)	.28	0.81 (0.68-0.98)	.03
	Male	858 (121)	0.74 (0.51-1.07)	.11	2217 (96)	0.93 (0.64-1.34)	.69	0.83 (0.64-1.08)	.16
	Female	745 (84)	0.70 (0.45-1.11)	.13	2360 (152)	0.84 (0.61-1.15)	.27	0.79 (0.61-1.03)	.08
	IGHG-high risk	410 (98)	0.82 (0.54-1.24)	.35	1313 (164)	0.81 (0.60-1.09)	.17	0.81 (0.64-1.04)	.09
	IGHG-moderate risk	294 (30)	0.87 (0.41-1.81)	.70	919 (40)	0.98 (0.52-1.82)	.94	0.93 (0.58-1.50)	.76
	IGHG-low risk	507 (35)	0.22 (0.08-0.60)	.003	1738 (16)	0.91 (0.37-2.25)	.84	0.48 (0.25-0.95)	.03
	Anthracyclines only (no heart RT)	583 (62)	0.48 (0.27-0.87)	.02	1171 (62)	0.95 (0.60-1.49)	.81	0.74 (0.51-1.05)	.09
	Anthracyclines (with or without heart RT)	1221 (154)	0.69 (0.50-0.96)	.03	2462 (176)	0.86 (0.66-1.13)	.29	0.79 (0.64-0.97)	.03
	Heart RT only (no anthracyclines)	382 (51)	0.87 (0.48-1.56)	.64	2115 (72)	0.94 (0.61-1.45)	.77	0.91 (0.64-1.30)	.61
	Heart RT (with or without anthracyclines)	1020 (143)	0.83 (0.60-1.15)	.27	3406 (186)	0.91 (0.70-1.18)	.46	0.87 (0.71-1.08)	.21
BAG3 (rs2234962)	All	1602 (205)	0.73 (0.55-0.96)	.03	4575 (248)	0.83 (0.65-1.07)	.15	0.79 (0.65-0.95)	.01
	Male	858 (121)	0.68 (0.47-0.97)	.04	2216 (96)	0.71 (0.47-1.06)	.10	0.69 (0.53-0.91)	.007
	Female	744 (84)	0.81 (0.52-1.26)	.34	2359 (152)	0.91 (0.66-1.24)	.54	0.87 (0.67-1.13)	.29
	IGHG-high risk	410 (98)	0.59 (0.37-0.93)	.02	1312 (164)	0.83 (0.61-1.13)	.24	0.74 (0.57-0.96)	.02
	IGHG-moderate risk	294 (30)	0.77 (0.37-1.58)	.47	919 (40)	1.06 (0.59-1.93)	.84	0.93 (0.59-1.48)	.76
	IGHG-low risk	505 (35)	0.73 (0.38-1.42)	.35	1737 (16)	0.96 (0.38-2.40)	.93	0.80 (0.47-1.37)	.42
	Anthracyclines only (no heart RT)	583 (62)	0.68 (0.40-1.15)	.15	1171 (62)	1.06 (0.65-1.71)	.82	0.86 (0.61-1.23)	.42
	Anthracyclines (with or without heart RT)	1222 (154)	0.70 (0.51-0.97)	.03	2461 (176)	0.85 (0.64-1.13)	.27	0.78 (0.63-0.97)	.03
	Heart RT only (no anthracyclines)	380 (51)	0.74 (0.42-1.30)	.29	2114 (72)	0.91 (0.58-1.42)	.67	0.84 (0.59-1.19)	.33
	Heart RT (with or without anthracyclines)	1019 (143)	0.73 (0.52-1.02)	.07	3404 (186)	0.83 (0.62-1.09)	.17	0.79 (0.63-0.97)	.03

CCM, cancer therapy-related cardiomyopathy; OR, odds ratio; CI, confidence interval; RT, radiotherapy; IGHG, International Late Effects of Childhood Cancer Guideline Harmonization Group; IGHG-high risk (cumulative anthracycline dose ≥250 mg/m^2, chest-directed radiotherapy ≥30 Gray [Gy], or a combination of cumulative anthracyclines ≥100 mg/m^2 and chest-directed radiotherapy ≥15 Gy); IGHG-moderate risk (100-249 mg/m^2 anthracyclines or chest-directed radiotherapy 15-29 Gy); IGHG-low risk (1-99 mg/m^2 anthracyclines and chest-directed radiotherapy <15 Gy)

© 2025 Neupane A et al. *JAMA Network Open*.

eTable 4. Cross-Sectional Association of *TTN* (rs3829746-C) and *BAG3* (rs2234962-C) With Echocardiographic Parameters (Baseline) in the St Jude Lifetime Cohort (SJLIFE)

Survivors	Echocardiographic parameters	SJLIFE EUR						SJLIFE AFR					
		BAG3 (rs2234962)			*TTN* (rs3829746)			*BAG3* (rs2234962)			*TTN* (rs3829746)		
		β*	SE**	*P*	β*	SE**	*P*	β*	SE**	*P*	β*	SE**	*P*
All	LV ejection fraction	0.86	0.27	.001	0.62	0.27	.02	2.68	1.74	.12	0.85	0.64	.19
	LV end-diastolic volume	-3.38	1.14	.003	-2.11	1.16	.07	1.73	7.03	.81	-2.04	2.60	.43
	LV end-systolic volume	-2.68	0.64	.003	-1.90	0.65	.003	-2.7	4.66	.56	-2.23	1.71	.19
	LV stroke volume	-0.90	0.66	.18	-0.30	0.67	.66	4.72	3.61	.19	-0.01	1.34	.99
	LV mass index	0.84	2.57	.75	-1.43	2.67	.59	-11.6	10.93	.29	-1.77	3.56	.62
	Global longitudinal peak strain	-0.3	0.12	.02	-0.31	0.13	.02	-1.18	0.81	.15	0.22	0.30	.45
	LV relative wall thickness	0	0	.69	0	0	.85	0.02	0.02	.53	-0.01	0.01	.24
Male	LV ejection fraction	0.99	0.36	.007	0.72	0.37	.05	3	2.81	.29	1.13	1.05	.29
	LV end-diastolic volume	-3.10	1.66	.06	-3.63	1.71	.03	-13.82	11.71	.24	-3.98	4.39	.37
	LV end-systolic volume	-3.05	0.95	.001	-2.94	0.98	<.001	-10.67	7.86	.18	-3.83	2.94	.20
	LV stroke volume	-0.41	0.97	.67	-0.84	1.00	.40	-3.15	5.78	.59	-0.15	2.16	.94
	LV mass index	-3.95	2.08	.06	1.34	2.15	.54	-10.86	23.63	.65	-6.01	6.84	.39
	Global longitudinal peak strain	-0.22	0.16	.18	-0.23	0.17	.17	-1.06	1.13	.35	-0.06	0.43	.89
	LV relative wall thickness	0	0	.77	0	0	.95	0.09	0.04	.02	-0.01	0.01	.46
Female	LV ejection fraction	0.74	0.40	.06	0.42	0.39	.29	1.21	2.34	.61	0.7	0.86	.42
	LV end-diastolic volume	-3.77	1.52	.01	0.32	1.51	.83	17.79	8.76	.045	-2.5	3.32	.45
	LV end-systolic volume	-2.30	0.81	.005	-0.29	0.81	.72	6.69	5.43	.22	-2.22	2.00	.27
	LV stroke volume	-1.49	0.89	.10	0.60	0.88	.50	11.31	4.98	.03	-0.78	1.90	.68
	LV mass index	4.94	4.47	.27	-3.19	4.65	.49	-2.15	15.50	.89	-5.03	5.02	.33
	Global longitudinal peak strain	-0.41	0.19	.03	-0.36	0.20	.07	-0.84	1.21	.49	0.12	0.44	.78
	LV relative wall thickness	0	0.01	.51	0	0.01	.81	-0.04	0.03	.25	0	0.01	.99

LV, left ventricular; β, beta coefficient; SE, standard error

*The beta coefficient represents the effect size of the independent SNV on the outcome variable, estimated using linear regression models. A positive beta indicates an increase in the outcome with an increase in the independent variable, while a negative beta suggests a decrease in the outcome. All analyses assumed an additive model of genetic inheritance, where each additional copy of the risk allele was modeled as having a consistent effect on the outcome.

**The standard error represents the precision of the beta estimate, quantifying the variability of the effect size across different samples.

© 2025 Neupane A et al. *JAMA Network Open*.

eTable 5. Longitudinal Association of *TTN* (rs3829746-C) and *BAG3* (rs2234962-C) With Echocardiographic Parameters (Baseline and Follow-Up Visits) in the St Jude Lifetime Cohort (SJLIFE)

Survivors	Echocardiographic parameters	SJLIFE EUR						SJLIFE AFR					
		BAG3 (rs2234962)			*TTN* (rs3829746)			*BAG3* (rs2234962)			*TTN* (rs3829746)		
		β*	SE**	*P*	β*	SE**	*P*	β*	SE**	*P*	β*	SE**	*P*
All	LV ejection fraction	0.72	0.22	.001	0.43	0.23	.06	1.45	1.57	.36	-0.03	0.58	.96
	LV end-diastolic volume	-3.61	1.09	<.001	-1.55	1.12	.17	0.59	7.01	.93	-1.25	2.58	.63
	LV end-systolic volume	-2.51	0.59	<.001	-1.32	0.61	.03	-1.38	4.32	.75	-0.79	1.59	.62
	LV stroke volume	-1.15	0.62	.06	-0.30	0.63	.64	2.15	3.56	.55	-0.66	1.31	.61
	LV mass index	1.12	2.48	.65	-1.14	2.53	.65	-13.91	12.11	.25	-1.28	3.84	.74
	Global longitudinal peak strain	-0.3	0.11	.006	-0.28	0.11	.01	-1.08	0.85	.21	0.40	0.32	.20
	LV relative wall thickness	0	0	.99	0	0	.59	0.01	0.02	.60	0	0.01	.56
Male	LV ejection fraction	0.95	0.30	.002	0.55	0.31	.08	1.14	2.36	.63	0.06	0.91	.95
	LV end-diastolic volume	-3.92	1.60	.01	-2.55	1.65	.12	-11.91	11.09	.28	-2.33	4.26	.58
	LV end-systolic volume	-3.07	0.89	<.001	-2.09	0.92	.02	-6.57	6.51	.31	-1.46	2.53	.56
	LV stroke volume	-0.97	0.90	.28	-0.63	0.93	.50	-5	5.68	.38	-1.03	2.18	.64
	LV mass index	-3.62	2.04	.08	1.39	2.06	.50	-22.09	25.05	.38	-2.22	7.35	.76
	Global longitudinal peak strain	-0.29	0.14	.04	-0.23	0.15	.11	-0.39	1.18	.74	0.25	0.46	.60
	LV relative wall thickness	0	0	.52	0	0	.84	0.04	0.03	.23	-0.01	0.01	.38
Female	LV ejection fraction	0.43	0.34	.20	0.24	0.34	.48	0.69	2.27	.76	-0.07	0.81	.93
	LV end-diastolic volume	-3.22	1.45	.03	0.01	1.46	.99	14.19	9.23	.12	-3.44	3.41	.31
	LV end-systolic volume	-1.83	0.76	.02	-0.18	0.76	.82	5.82	5.54	.29	-1.97	2.02	.33
	LV stroke volume	-1.34	0.82	.10	0.26	0.83	.76	8.45	4.98	.09	-1.89	1.83	.30
	LV mass index	5.53	4.32	.20	-3.33	4.43	.45	2.87	18.47	.88	-5.54	5.40	.31
	Global longitudinal peak strain	-0.29	0.18	.10	-0.32	0.18	.07	-0.99	1.31	.45	0.28	0.47	.55
	LV relative wall thickness	0	0	.68	0	0	.55	-0.03	0.03	.36	0.01	0.01	.58

LV, left ventricular; β, beta coefficient; SE, standard error

*The beta coefficient represents the effect size of the independent genetic variant on the outcome variable, estimated using a multivariable mixed-effects model. In this model, beta reflects the association between the genetic variant and the outcome, adjusted for fixed effects such as covariates and random effects (to account for within-subject variability or other random factors). A positive beta indicates that an increase in the genetic variable is associated with an increase in the outcome, while a negative beta suggests a decrease in the outcome. The model assumes an additive genetic inheritance model.

**The standard error represents the precision of the beta estimate, quantifying the variability of the effect size across different samples.

© 2025 Neupane A et al. *JAMA Network Open*.

eTable 6. Rare (Minor Allele Frequency [MAF] <1 × 10^{-4}, per gnomAD v4.1 Non-Finnish European [NFE] Population) Protein-Altering Variants (PAV) Within Familial Dilated Cardiomyopathy Genes, Identified in Childhood Cancer Survivors of European Ancestry

Chr	Position (hg38)	rsID	Genes	Minor allele	Major allele	Variation	MAF (gnomAD NFE)	Average PSI (GTeX)	*TTN* band
1	156114941	rs1329278578	*LMNA*	A	G	missense	1.47E-05		
1	156115105	rs899373360	*LMNA*	C	A	missense	1.47E-05		
1	156115208	rs1060502216	*LMNA*	C	A	missense	1.47E-05		
1	156126893	rs752549023	*LMNA*	T	C	missense	1.47E-05		
1	156126900	rs148559653	*LMNA*	G	A	missense	2.94E-05		
1	156134454	rs267607626	*LMNA*	T	C	missense	1.47E-05		
1	156134823	rs370134870	*LMNA*	T	C	missense	4.41E-05		
1	156134824	rs780066296	*LMNA*	A	G	missense	1.47E-05		
1	156134891	rs763625309	*LMNA*	A	G	missense	5.88E-05		
1	156134914	rs397517907	*LMNA*	T	C	missense	2.94E-05		
1	156135224	rs765241364	*LMNA*	G	A	missense	2.94E-05		
1	156135260	rs769210828	*LMNA*	T	C	missense	4.41E-05		
1	156135268	rs59885338	*LMNA*	T	C	missense	2.94E-05		
1	156135916	rs267607574	*LMNA*	A	G	missense	1.47E-05		
1	156136240	rs267607561	*LMNA*	T	C	missense	1.47E-05		
1	156136311	rs755686359	*LMNA*	T	C	missense	5.88E-05		
1	156136335	rs373584456	*LMNA*	T	C	missense	1.47E-05		
1	156136359	rs150840924	*LMNA*	T	C	missense	0		
1	156136374	rs121912493	*LMNA*	A	G	missense	2.94E-05		
1	156137141	rs878855233	*LMNA*	C	A	missense	5.88E-05		
1	156138821	rs911500699	*LMNA*	T	C	stop-gained	2.94E-05		
1	156138894	rs896304251	*LMNA*	T	G	missense	2.94E-05		
1	156138902	rs955331662	*LMNA*	G	C	missense	1.47E-05		
1	201359177	rs764761207	*TNNT2*	A	G	missense	0		
1	201361272	rs373069229	*TNNT2*	A	G	missense	1.47E-05		
1	201361274	rs730881113	*TNNT2*	T	C	missense	7.35E-05		
1	201362019	rs150008205	*TNNT2*	T	C	missense	1.47E-05		
1	201363391	rs397516469	*TNNT2*	A	G	stop-gained	1.47E-05		
1	201365656	rs397516450	*TNNT2*	C	T	missense	8.82E-05		
2	178532522	rs1689606484	*TTN*	C	CG	frameshift	1.47E-05	1	M-band
2	178575970	rs781540455	*TTN*	A	G	stop-gained	1.47E-05	0.95	A-band
2	178706629	rs746721983	*TTN*	A	G	stop-gained	0	0.89	I-band
2	178745884	rs148430495	*TTN*	A	C	stop-gained	8.84E-05	0.89	
2	178746079	rs201273719	*TTN*	A	G	stop-gained	2.94E-05	0.89	
2	178750594	rs147087155	*TTN*	A	G	stop-gained	1.47E-05	0.89	
3	52451415	rs730881061	*TNNC1*	C	T	missense	2.94E-05		
6	7574797	rs774514264	*DSP*	C	T	splice-donor	2.94E-05		
10	119651746	rs771609568	*BAG3*	G	C	missense	2.94E-05		

© 2025 Neupane A et al. *JAMA Network Open*.

10	119651808	rs747820097	*BAG3*	T	C	missense	4.41E-05
10	119669881	rs387906874	*BAG3*	T	C	missense	7.35E-05
10	119669985	rs566015884	*BAG3*	G	C	missense	0
10	119670088	rs780169403	*BAG3*	A	G	missense	0
10	119670122	rs770678896	*BAG3*	A	G	missense	1.47E-05
10	119672255	rs200479768	*BAG3*	T	C	missense	7.35E-05
10	119672297	rs1847161262	*BAG3*	G	T	missense	1.47E-05
10	119672426	rs149517238	*BAG3*	G	C	missense	8.82E-05
10	119672445	rs1415830597	*BAG3*	G	A	missense	4.41E-05
10	119672490	rs369947845	*BAG3*	G	A	missense	7.35E-05
10	119672574	rs372970842	*BAG3*	A	G	missense	8.82E-05
10	119672601	rs375650805	*BAG3*	T	C	missense	1.47E-05
10	119672619	rs368866313	*BAG3*	T	C	missense	5.88E-05
10	119676672	rs876657745	*BAG3*	A	G	missense	1.47E-05
10	119677132	rs777325497	*BAG3*	T	G	missense	4.41E-05
14	23415035	rs149193520	*MYH7*	G	A	missense	2.94E-05
14	23415047	rs727503242	*MYH7*	A	G	missense	5.88E-05
14	23415651	rs193922390	*MYH7*	T	C	missense	4.41E-05
14	23415832	rs397516233	*MYH7*	A	C	missense	1.47E-05
14	23416089	rs1306407579	*MYH7*	C	T	missense	5.88E-05
14	23416240	rs750987717	*MYH7*	T	C	missense	1.47E-05
14	23417309	rs876661373	*MYH7*	T	C	missense	4.41E-05
14	23417555	rs780625785	*MYH7*	T	C	missense	2.94E-05
14	23418348	rs797045097	*MYH7*	T	C	missense	0
14	23419557	rs747308839	*MYH7*	T	C	missense	5.88E-05
14	23420164	rs730880905	*MYH7*	T	C	missense	1.47E-05
14	23420234	rs193922388	*MYH7*	T	C	missense	1.48E-05
14	23420993	rs367546859	*MYH7*	T	C	missense	4.41E-05
14	23422315	rs1159928168	*MYH7*	T	G	missense	2.94E-05
14	23424148	rs397516161	*MYH7*	C	T	missense	1.47E-05
14	23425345	rs376754645	*MYH7*	T	C	missense	4.41E-05
14	23427283	rs369935820	*MYH7*	A	G	missense	4.41E-05
14	23429028	rs752349938	*MYH7*	A	G	missense	4.41E-05
14	23429037	rs730880870	*MYH7*	T	C	missense	5.88E-05
14	23433218	rs730880830	*MYH7*	T	C	missense	1.47E-05
14	23433618	rs376160714	*MYH7*	T	C	missense	4.41E-05
14	23433684	rs45511396	*MYH7*	A	G	missense	4.41E-05
17	39665382	rs778568339	*TCAP*	GCGAGGTGT	G	frameshift	1.47E-05
17	39665813	rs775636212	*TCAP*	T	C	missense	1.47E-05
17	39665814	rs552865793	*TCAP*	A	G	missense	1.47E-05
17	39665831	rs572836774	*TCAP*	T	C	missense	8.82E-05
17	39665991	rs1324156287	*TCAP*	G	A	missense	4.41E-05
17	39666026	rs45509691	*TCAP*	G	C	missense	4.41E-05

© 2025 Neupane A et al. *JAMA Network Open*.

The types of variants considered for this study were based on the following criteria: all protein-altering variants for *BAG3*, *LMNA*, *TCAP*, *TNNC1*, and *TNNT2*; missense variants and in-frame insertions or deletions for *MYH7*; and frameshift, stop-gained, splice-donor, and splice-acceptor variants for *DSP*, *SCN5A*, and *TTN*. Although all nine genes were initially considered for the analysis, no survivor carried rare PAVs within *SCN5A*.

© 2025 Neupane A et al. *JAMA Network Open*.

eTable 7. Rare (Minor Allele Frequency [MAF] <1 × 10^{-4}, per gnomAD v4.1 African Population) Protein-Altering Variants (PAVs) Within Familial Dilated Cardiomyopathy Genes, Identified in Childhood Cancer Survivors of African Ancestry

Chr	Position (hg38)	rsID	Genes	Minor allele	Major allele	Variation	MAF (gnomAD v4.1 African)	Average PSI (GTEx)	*TTN* band
1	156136245	rs374726751	*LMNA*	T	C	missense	4.82E-05		
1	156138822	rs1001682649	*LMNA*	A	G	missense	0		
1	201359220	rs141121678	*TNNT2*	T	C	missense	0		
1	201359224	rs147940106	*TNNT2*	T	C	missense	0		
2	178745811	rs1331792718	*TTN*	GCTGT	G	frameshift	4.83E-05	0.89	
10	119670056	rs876657747	*BAG3*	T	C	missense	4.82E-05		
14	23415773	rs779978846	*MYH7*	C	G	missense	7.24E-05		
14	23417179	rs1445128471	*MYH7*	T	A	missense	0		
14	23418385	rs397516198	*MYH7*	T	C	missense	2.41E-05		
14	23421000	rs375323916	*MYH7*	A	C	missense	4.83E-05		
14	23423913	rs141191984	*MYH7*	G	C	missense	4.83E-05		

The types of variants considered for this study were based on the following criteria: all protein-altering variants for *BAG3*, *LMNA*, *TCAP*, *TNNC1*, and *TNNT2*; missense variants and in-frame insertions or deletions for *MYH7*; and frameshift, stop-gained, splice-donor, and splice-acceptor variants for *DSP*, *SCN5A*, and *TTN*. Although all nine genes were initially considered for the analysis, no survivor carried rare PAVs within *TCAP*, *TNNCI*, *DSP*, *SCN5A*.

© 2025 Neupane A et al. *JAMA Network Open*.

eTable 8. Association of Rare Protein-Altering Variants (PAVs) in Familial DCM-Related Genes From the General Population, With Late-Onset CCM Risk in Long-Term Childhood Cancer Survivors of European Ancestry From the St Jude Lifetime Cohort (SJLIFE) and Childhood Cancer Survivor Study (CCSS)

		SJLIFE European ancestry (205 with CCM and 1400 without CCM)					CCSS European ancestry (241 with CCM and 4217 without CCM)					Meta-analysis*	
**Genes**	**Total number of variants**	**Total number of carriers**	**n carriers with CCM (% [95% CI])**	**n carriers without CCM (% [95% CI])**	**OR (95% CI)**	***P***	**Total number of carriers**	**n carriers with CCM (% [95% CI])**	**n carriers without CCM (% [95% CI])**	**OR (95% CI)**	***P***	**OR (95% CI)**	***P***
BAG3	16	8	1 (0.49 [0.01-2.69])	7 (0.5 [0.2-1.03])	0.98 (0.02-7.66)	>.99	12	0 (0 [0-1.52])	12 (0.28 [0.15-0.5])	0 (0-6.32)	>.99	0.56 (0.07, 4.38)	.89
DSP	1	1	1 (0.49 [0.01-2.69])	0 (0 [0-0.26])	Inf (0.18-Inf)	.13	0	0 (0 [0-1.52])	0 (0 [0-0.09])	0 (0-6.32)	>.99	ND	ND
LMNA	23	12	4 (1.95 [0.53-4.92])	8 (0.57 [0.25-1.12])	3.46 (0.76-13.05)	.06	18	0 (0 [0-1.52])	18 (0.43 [0.25-0.67])	0 (0-3.99)	.62	1.76 (0.59, 5.26)	.51
MYH7	22	11	1 (0.49 [0.01-2.69])	10 (0.71 [0.34-1.31])	0.68 (0.02-4.84)	>.99	16	2 (0.83 [0.1-2.97])	14 (0.33 [0.18-0.56])	2.51 (0.28-11.03)	.21	1.36 (0.41, 4.52)	.87
TCAP	6	1	0 (0 [0-1.78])	1 (0.07 [0-0.4])	0 (0-265.24)	>.99	10	0 (0 [0-1.52])	10 (0.24 [0.11-0.44])	0 (0-7.84)	>.99	ND	ND
TNNC1	1	1	0 (0 [0-1.78])	1 (0.07 [0-0.4])	0 (0-265.24)	>.99	1	0 (0 [0-1.52])	1 (0.02 [0-0.13])	0 (0-675.23)	>.99	ND	ND
TNNT2	6	5	0 (0 [0-1.78])	5 (0.36 [0.12-0.83])	0 (0-7.48)	>.99	2	0 (0 [0-1.52])	2 (0.05 [0.01-0.17])	0 (0-93.54)	>.99	ND	ND
TTN (PSI >0.82)	6	3	0 (0 [0-1.78])	3 (0.21 [0.04-0.62])	0 (0-16.57)	>.99	6	0 (0 [0-1.52])	6 (0.14 [0.05-0.31])	0 (0-14.92)	>.99	ND	ND
TTN (PSI >0.82 and within A band)	1	1	0 (0 [0-1.78])	1 (0.07 [0-0.4])	0 (0-265.24)	>.99	0	0 (0 [0-1.52])	0 (0 [0-0.09])	0 (0-14.92)	>.99	ND	ND
BAG3 + *TTN* (PSI >0.82)	22	11	1 (0.49 [0.01-2.69])	10 (0.71 [0.34-1.31])	0.68 (0.02-4.84)	>.99	18	0 (0 [0-1.52])	18 (0.43 [0.25-0.67])	0 (0-3.99)	.62	0.39 (0.05, 2.94)	.55
BAG3 + *TTN* (PSI >0.82 and within A band)	17	9	1 (0.49 [0.01-2.69])	8 (0.57 [0.25-1.12])	0.85 (0.02-6.42)	>.99	12	0 (0 [0-1.52])	12 (0.28 [0.15-0.5])	0 (0-6.32)	>.99	0.52 (0.07, 4.02)	.81
ALL protein altering variants including *TTN* (PSI >0.82) variants	81	42	7 (3.41 [1.38-6.91])	35 (2.5 [1.75-3.46])	1.38 (0.51-3.21)	.48	61	2 (0.83 [0.1-2.97])	59 (1.4 [1.07-1.8])	0.59 (0.07-2.25)	.77	1.05 (0.52, 2.11)	.90
ALL protein altering variants including *TTN* (PSI >0.82 and within A band) variants	76	40	7 (3.41 [1.38-6.91])	33 (2.36 [1.63-3.29])	1.46 (0.54-3.43)	.34	56	2 (0.83 [0.1-2.97])	54 (1.28 [0.96-1.67])	0.65 (0.08-2.47)	.77	1.12 (0.56, 2.28)	.89

ND, not-determined; CCM, cancer therapy-induced cardiomyopathy; CI, confidence interval

The types of variants considered for this study were based on the following criteria: all protein-altering variants for *BAG3*, *LMNA*, *TCAP*, *TNNC1*, and *TNNT2*; missense variants and in-frame insertions or deletions for *MYH7*; and frameshift,

© 2025 Neupane A et al. *JAMA Network Open*.

stop-gained, splice-donor, and splice-acceptor variants for *DSP*, *SCN5A*, and *TTN*. Although all nine genes were initially considered for the analysis, no survivor carried rare PAVs within *SCN5A*.

*Meta-analysis of results from the SJLIFE and CCSS cohorts were conducted using Cochran–Mantel–Haenszel two-by-two test.

© 2025 Neupane A et al. *JAMA Network Open*.

eTable 9. Association of Rare Protein-Altering Variants (PAVs) Within Familial DCM-Related Genes From the General Population in Long-Term Survivors of Childhood Cancer From the St Jude Lifetime Cohort of African Ancestry

Carriers of variants	African ancestry (37 with CCM and 201 without CCM)					
	Total number of variants	Total number of carriers	n carriers with CCM (% [95% CI])	n carriers without CCM (% [95% CI])	OR (95% CI)	***P***
BAG3	1	1	0 (0 [0-9.49])	1 (0.50 [0.01-2.74])	0 (0.00-211.17)	>.99
LMNA	2	2	0 (0 [0-9.49])	2 (1.00 [0.12-3.55])	0 (0.00-29.18)	>.99
MYH7	5	5	1 (2.70 [0.07-14.16])	4 (1.99 [0.54-5.02])	1.37 (0.03-14.33)	.57
TNNT2	2	2	0 (0 [0-9.49])	2 (1.00 [0.12-3.55])	0 (0.00-29.18)	>.99
TTN (PSI > 0.82)	1	2	1 (2.70 [0.07-14.16])	1 (0.50 [0.01-2.74])	5.49 (0.07-436.54)	.29
BAG3 + *TTN* (PSI >0.82)	2	3	1 (2.70 [0.07-14.16])	2 (1.00 [0.12-3.55])	2.75 (0.05-54.04)	.40
BAG3 + *TTN* (PSI >0.82 and within A band)	1	1	0 (0 [0-9.49])	1 (0.50 [0.01-2.74])	0 (0-211.17)	>.99
ALL protein altering variants including *TTN* (PSI >0.82) variants	11	12	2 (5.41 [0.66-18.19])	10 (4.98 [2.41-8.96])	1.09 (0.11-5.45)	>.99
ALL protein altering variants including *TTN* (PSI >0.82 and within A band) variants	10	10	1 (2.70 [0.07-14.16])	9 (4.48 [2.07-8.33])	0.59 (0.01-4.52)	>.99

ND, not-determined; CCM, cancer therapy-induced cardiomyopathy; CI, confidence interval

The types of variants considered for this study were based on the following criteria: all protein-altering variants for *BAG3*, *LMNA*, *TCAP*, *TNNC1*, and *TNNT2*; missense variants and in-frame insertions or deletions for *MYH7*; and frameshift, stop-gained, splice-donor, and splice-acceptor variants for *DSP*, *SCN5A*, and *TTN*. Although all nine genes were initially considered for the analysis, no survivors carried rare PAVs within *TCAP*, *TNNCI*, *DSP*, *SCN5A*.

© 2025 Neupane A et al. *JAMA Network Open*.

eTable10. Association Of Rare Protein-Altering Variants (PAVs) in Familial DCM-Related Genes From the General Population, With Late-Onset CCM Risk in Long-Term Childhood Cancer Survivors of European Ancestry from the St Jude Lifetime Cohort (SJLIFE) and Childhood Cancer Survivor Study (CCSS), With Additional Adjustment for Follow-Up Duration, Coronary Artery Disease (CAD), and Cardiovascular Risk Factors (CVRFs: hypertension, diabetes, dyslipidemia and obesity)

**Carriers of variants**	**Main model**		**Main model + follow-up duration + CAD + CVRFs**	
	**OR (95% CI)**	***P***	**OR (95% CI)**	***P***
ALL protein altering variants including *TTN* (PSI > 0.82) variants	1.54 (0.60-3.49)	.33	1.61 (0.61-3.76)	.30
ALL protein altering variants including *TTN* (PSI > 0.82 and within A band) variants	1.56 (0.60-3.55)	.32	1.62 (0.61-3.79)	.30

OR, odds ratio; CI, confidence interval

The types of variants considered for this study were based on the following criteria: all protein-altering variants for *BAG3*, *LMNA*, *TCAP*, *TNNC1*, and *TNNT2*; missense variants and in-frame insertions or deletions for *MYH7*; and frameshift, stop-gained, splice-donor, and splice-acceptor variants for *DSP*, *SCN5A*, and *TTN*. Although all nine genes were initially considered for the analysis, no survivor carried rare PAVs within *SCN5A*, and analyses were limited to those with at least five survivors carrying rare PAVs.

Analyses in the Main model were adjusted for sex, age at diagnosis, age at last contact, cumulative anthracycline dose, average heart radiation dose, treatment era and top ten principal components based on the genotype data

Since fewer than 5 survivors with CCM carried rare PAVs in each gene, separate analyses for each gene could not be conducted.

© 2025 Neupane A et al. *JAMA Network Open*.
