# Supplementary material for: TTN and BAG3 in Cancer Therapy–Related Cardiomyopathy Among Long-Term Survivors of Childhood Cancer
Source: JAMA Netw Open. 2025 Jun 20;8(6):e2515793. doi: 10.1001/jamanetworkopen.2025.15793 (PMC12181783; doi:10.1001/jamanetworkopen.2025.15793)
Supplement: Supplement 2. — Data Sharing Statement [file jamanetwopen-e2515793-s002.pdf]

# Data Sharing Statement

Neupane. TTN and BAG3 in Cancer Therapy–Related Cardiomyopathy Among Long-Term Survivors of Childhood Cancer. *JAMA Netw Open*. Published June 13, 2025. doi:10.1001/jamanetworkopen.2025.15793

**Data**
**Data available:** No

**Additional Information**
**Explanation for why data not available:** Genotype and phenotype data for SJLIFE and CCSS survivors are accessible through the St Jude Cloud (https://stjude.cloud) or through the database of Genotypes and Phenotypes (dbGaP accession number: phs001327.v2.p1). For CCSS, additional phenotype data may be requested through https://ccss.stjude.org/.
